# Hierarchical imputation of categorical variables in the presence of systematically and sporadically missing data

**DOI:** 10.1017/rsm.2025.10017

**Published:** 2025-06-10

**Authors:** Shahab Jolani

**Affiliations:** Department of Methodology and Statistics, Care and Public Health Research Institute (CAPHRI), Maastricht University, Maastricht, The Netherlands

**Keywords:** individual participants data (IPD) meta-analysis, missing data, multilevel model, multiple imputation (MI), multivariate imputation using chained equations (MICE)

## Abstract

Modern quantitative evidence synthesis methods often combine patient-level data from different sources, known as individual participants data (IPD) sets. A specific challenge in meta-analysis of IPD sets is the presence of systematically missing data, when certain variables are not measured in some studies, and sporadically missing data, when measurements of certain variables are incomplete across different studies. Multiple imputation (MI) is among the better approaches to deal with missing data. However, MI of hierarchical data, such as IPD meta-analysis, requires advanced imputation routines that preserve the hierarchical data structure and accommodate the presence of both systematically and sporadically missing data. We have recently developed a new class of hierarchical imputation methods within the MICE framework tailored for continuous variables. This article discusses the extensions of this methodology to categorical variables, accommodating the simultaneous presence of systematically and sporadically missing data in nested designs with arbitrary missing data patterns. To address the challenge of the categorical nature of the data, we propose an accept–reject algorithm during the imputation process. Following theoretical discussions, we evaluate the performance of the new methodology through simulation studies and demonstrate its application using an IPD set from patients with kidney disease.

## Highlights

### What is already known?

- Systematically and sporadically missing data are very common in individual participant data meta-analysis (IPD-MA).
- Multiple imputation of missing data in IPD-MA should account for clustering and heterogeneity between studies.
- Multilevel imputation methods are available for continuous variables with both systematically and sporadically missing data.

### What is new?

- We develop a new class of imputation methods for categorical variables in IPD-MA while addressing the challenge of simultaneous presence of systematically and sporadically missing data.

### Potential impact for RSM readers

- Imputation of missing values in IPD-MA allows using full data potential which can provide additional insight for evidence synthesis.
- The proposed imputation methodology can directly be applied to other settings of clustered (or multilevel) data such as longitudinal studies.

## 1 Introduction

Quantitative evidence synthesis is an important aspect of contemporary clinical research and medical decision making. It is widely used to summarize the effectiveness of medical interventions,^1^ the accuracy of diagnostic tests,^2^ the association of prognostic factors,^3^ or even the performance of published prediction models.^4^ Historically, evidence synthesis originates from the desire to summarize the results from multiple related studies, often through meta-analysis techniques that aggregate published data (e.g., estimates of relative treatment effect) into a weighted average. However, there is a growing trend toward combining patient-level data from multiple studies and performing a so-called individual participant data meta-analysis (IPD-MA).^5^ ^,^ ^6^

IPD-MA represents a new era in evidence synthesis, offering several advantages over traditional meta-analyses that are solely based on aggregate data. Most notably, the use of IPD allows for more precise tailoring of diagnostic strategies, identification of risk and prognostic factors, and personalizing risk prediction.^7^ Moreover, pooling of IPD is particularly sui3 for identifying modifiers of relative treatment effect^8^ and developing models to predict absolute outcome risk or absolute treatment effects in individual patients.^9^

A key challenge in combining multiple sources of IPD is the presence of between-study heterogeneity. Briefly, this implies that studies differ in aspects such as outcome occurrence (baseline risk) or the magnitude of predictor-outcome associations. Such heterogeneity can arise from differences in participant eligibility criteria, variable and outcome definitions, measurement methods, or treatment protocols across studies. The presence of between-study heterogeneity is an important concern in any meta-analysis, as it may substantially affect the interpretation of pooled summary estimates.^10^

Another common issue in meta-analysis of IPD is the presence of missing data. This situation typically occurs when variables of interest have not been measured in one or more studies (resulting in completely or systematically missing) or in some participants within studies (resulting in partially or sporadically missing). For example, in the GREAT Network study,^11^ the biomarker *brain natriuretic peptide* (BNP) was used to explain left ventricular ejection fraction. However, BNP is a relatively recent technique which was unavailable (thus, systematically missing), in several studies within the IPD-MA of the GREAT study. While it is possible to restrict the analysis to participants with complete data, the potential loss of efficiency and validity is generally undesirable.^12^ Therefore, it is generally recommended to adopt multiple imputation (MI)^13^ methods when one or more studies are affected by missing data. Briefly, MI generates multiple versions of the original dataset(s) by replacing missing values with imputations that are based on observed data. A common approach is to use chained equations to impute each variable sequentially, conditional on the other variables.^14^ ^,^ ^15^ This method allows for a great deal of flexibility as each variable (e.g., binary, ordinal, continuous, etc.) can be imputed using an appropriate functional form, such as logistic or probit models for binary variables (see van Buuren ^16^). Although MI can be relatively straightforward to implement, it requires careful consideration when participants are clustered within different studies or centers. Imputation models should account for variability within and across studies; otherwise, the imputed values and their uncertainty may no longer be valid.^17^ ^,^ ^18^

For clustered data such as IPD, the multivariate imputation using chained equations (MICE) algorithm naturally extends to multilevel settings. This requires specifying conditional imputation models that incorporate random effects, known as *multilevel* or *hierarchical imputation* models. Although several multilevel imputation methods (MLMIs) have been proposed (for a recent overview, see Audigier et al.^19^) they have at least two limitations: (i) they are primarily developed for continuous (and normally distributed) variables and (ii) they are not designed to address the combined presence of sporadically and systematically missing data.

Several multilevel imputation routines are available for continuous variables within the MICE framework. Van Buuren^20^ and Yucel et al.,^21^ among others, developed multilevel imputation approaches based on linear mixed-effects models to handle sporadically missing data in continuous variables. While these methods effectively account for the multilevel structure of the data, they are not designed to handle systematically missing data. Further, Resche-Rigon and White^22^ and Jolani^23^ developed multilevel imputation approaches that address both systematically and sporadically missing data, but these are limited to continuous variables. In addition, Resche-Rigon and White^24^ proposed a two-stage multilevel imputation approach for systematically and sporadically missing data in continuous variables.

For categorical data, MLMIs are scarce and primarily limited to binary variables. Within the MICE framework, Yucel et al.^21^ developed a MLMIs for binary variables using the generalized linear mixed-effects models. However, this approach only addresses sporadically missing data and is not suitable for systematically missing data. Conversely, Jolani et al.^25^ proposed a general class of MLMIs for categorical variables with systematically missing data, but their method does not accommodate sporadically missing data. Motivated by the one-stage approach of Jolani et al.^25^ and two-stage approach of Resche-Rigon and White,^24^ Audigier et al.^19^ suggested treating sporadically missing values in binary variables as systematically missing and imputing them accordingly using the one-stage or two-stage multilevel approaches, respectively.

The combined presence of systematically and sporadically missing data in categorical variables is a common challenge in IPD-MA. To date, the MICE framework still lacks principled MLMIs that address both systematically and sporadically missing data in categorical variables. To bridge this gap, we propose a new class of multilevel imputation methodologies for IPD-MA and beyond, specifically designed to address the challenges posed by both systematically and sporadically missing data in categorical variables.

This manuscript is organized as follows. Section 2 introduces an innovative multilevel imputation methodology for categorical variables by utilizing an accept–reject sampling procedure. We mainly focus on the development of MLMIs for a broad class of families, including models for binary and count variables. Section 3 evaluates the performance of the proposed methodologies through extensive simulation studies across a wide range of scenarios, including varying degrees of between-study heterogeneity. Section 4 demonstrates the implementation of the new methodologies in an IPD-MA of chronic kidney disease (CKD) patients. Section 5 concludes with a discussion and final remarks.

## 2 Methods

### 2.1 Analysis model for IPD-MA

Suppose we aim to perform an IPD-MA from *n* studies. Let 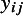 denote a value of the random variable *Y* for subject 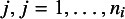 in study 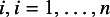 . We assume the following generalized linear mixed-effects model for the IPD set: 
(2.1)

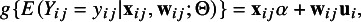

where 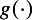 denotes a link function, 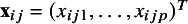 is a 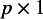 vector of potential predictors of outcome 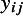 , 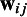 is typically a subset of vector 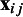 , 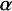 is a vector of fixed-effects parameters, and 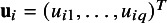 is a 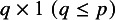 vector of random effects following a multivariate normal distribution, 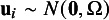 with a 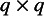 covariance matrix 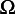 . We denote the diagonal elements of 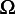 with 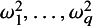 and its off-diagonal elements with 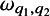 , 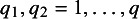 and 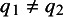 . We refer to such variances and covariances as random-effects parameters, and 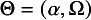 . Furthermore, we assume that the vectors 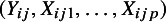 for all 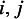 are independent and identically distributed and that the vectors 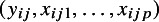 are their realizations.

The analysis model 2.1 encompasses a general family of distributions. For continuous outcome 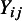 , for instance, the link function is identity ( 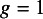 ) so we use 

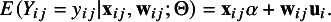

For binary outcome 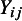 , as another example, the link function is logit ( 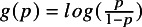 ) and the analysis model 2.1 is represented by 

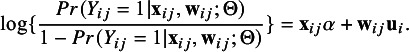



### 2.2 Multilevel MI in MICE

MICE is blind to the role of variables in the analysis, and each variable is imputed in turn conditional on the other variables. Let 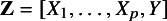 denote a full data matrix containing a set of explanatory variables 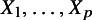 and outcome *Y*, and 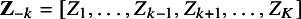 . Without loss of generality, we assume that each column of 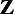 has some missing entries. The MICE (or fully conditional specification) approach specifies an appropriate conditional regression model for each variable (i.e., each column of 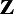 ) given the other variables, that is, 

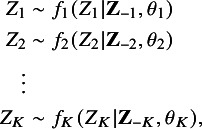

where 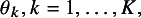 denotes the unknown parameters of each conditional distribution. These conditional models are the basis for approximating the posterior predictive distribution of the missing data. To account for the hierarchical data structure (i.e., individuals within studies), we apply a linear mixed-effects model for continuous variables, a logistic mixed-effects model for binary variables, a Poisson mixed-effects model for count variables, and so on. The set of specified conditional multilevel models forms a cycle, a few of which typically should be repeated successively to achieve an adequate approximation to the marginal posterior predictive distribution of the missing data. Specifically, missing values of 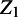 are imputed conditional on the other variables. Subsequently, missing values of 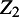 are imputed using the recent imputations of 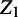 and other variables. The imputed values from the last cycle are eventually considered as one set of imputations. Replicating the whole process multiple times produces MI sets.

We throughout assume the missing data mechanism is missing at random (MAR)^26^ implying that the probability that any data value is missing may depend on quantities that are observed but not quantities that are missing. Therefore, the imputation model that we focus on would be appropriate under missing completely at random (MCAR) or MAR assumption.

### 2.3 Multilevel imputation of a single variable

Because MICE is implemented on a variable-by-variable basis, we focus on the imputation of a single (categorical) variable in this section. For subject *j* in study *i*, suppose 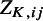 represents the *K*th (last) incomplete variable in the data matrix 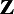 . We define the following multilevel imputation model for 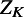 , which is a generalized linear mixed-effects model conditional on the remaining 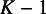 variables 
(2.2)

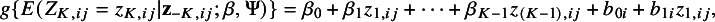

where *g* is the link function and 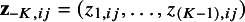 . For brevity, we assume a random intercept and a random slope model for 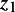 only in the above imputation model, so 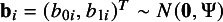 for 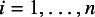 , where 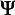 is a 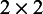 covariance matrix containing the random-effects parameters. The fixed-effects parameters are denoted by 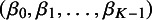 in the imputation model 2.2. Following Rubin^13^, the formal procedure to obtain imputations for 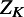 consists of the following steps:

1. Estimating the parameters 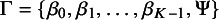 altogether with the random effects 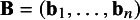 in model 2.2 using the observed data.
2. Drawing 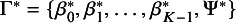 and subsequently 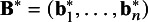 from their observed-data posterior distributions.
3. Imputing missing values of 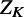 from the conditional predictive distribution 

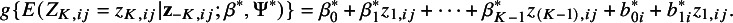

Although the implementation of step (1) is rather straightforward, drawing the parameters from the posterior distributions in step (2) is cumbersome as the unconditional distributions for these parameters cannot generally be obtained in closed form. Thus, Markov chain Monte Carlo methods are typically employed by combining steps (1) and (2) to estimate and obtain random draws of 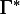 and 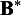 (see, among others, Drechsler^27^). Such iterative algorithms are, however, unattractive within the MICE framework because a Gibbs sampler needs to be iterated within each conditional model of a cycle. Jolani et al.^25^ proposed a simplification over the full Gibbs sampler that relies on the conditional independence between 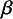 and 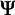 and requires no iteration. In short, inference about 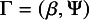 can be separated into two conditionally independent parts assuming that the random effects 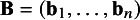 are known. Here, standard routines for the (generalized) linear mixed-effects models (e.g., the *glmer* function in the R package 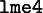 ) are used to estimate the parameters in step (1). Random draws of 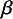 and 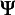 are then obtained independently conditional of the estimated random effects 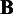 from step (1) (see Jolani^23^ for details).

This imputation methodology was originally developed by Jolani et al.^25^ for any systematically missing variable where the random effects 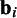 are drawn from the unconditional (prior) distribution 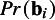 . Jolani^23^ further extended the proposed imputation methodology to systematically and sporadically missing continuous variables. Extensions to categorical variables with sporadically missing data are an uneasy task because the conditional posterior distribution of random effects does not have a standard form as opposed to continuous variables. To elaborate on this point, consider an incomplete variable *Z*. For study *i* with sporadically missing values, we need to make a draw from the posterior distribution 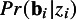 , which can be approximated by 
(2.3)

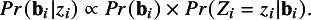

From model 2.2, we know that 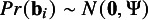 . If *Z* is normally distributed (and thus continuous), it follows that 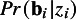 is normally distributed too, and so a random draw of 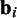 can easily be obtained conditional on 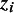 . However, it is difficult to simulate directly from this distribution if *Z* is categorical (e.g., binary). Therefore, we propose an accept–reject sampling method, ^28^ to draw a random value 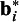 from the posterior distribution 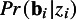 .

The accept–reject sampling method involves obtaining draws from a proposal density (which is easier to sample from) until a draw satisfies a particular condition. We choose 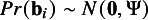 as a proposal density, from which samples can easily be drawn. The method then requires the ratio of the target density (i.e., 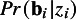 ) to the proposal density be bounded above a constant quantity *M*. It is easy to show that this ratio is proportional to 

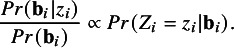

Following Robert and Casella,^28^ the bound *M* can be taken to be the likelihood function in equation (2.3) evaluated at the maximum likelihood estimates. The algorithm is then completed when we sample 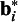 from the proposal density and *U* from the uniform distribution on ( 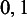 ). The drawn sample 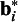 is accepted if 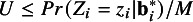 . Otherwise, a new pair ( 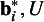 ) is drawn. After completing the above steps (1) and (2), imputations are obtained in step (3) using an appropriate generalized mixed-effects model.

The proposed accept–reject method is general and can be applied to any categorical variables as long as these include the family of generalized linear mixed-effects models. Also, it should be emphasized that, for a given incomplete variable, the proposed accept–reject sampling method is required for studies with sporadically missing data. For studies with systematically missing data, the random effects 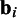 are drawn from the unconditional distribution 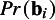 (see Jolani et al.^25^). As a showcase, we provide computational details of the proposed imputation methodology for a count variable with systematically and sporadically missing data.

Example 2.1. Multilevel imputation of a count variable

For simplicity suppose the data matrix 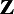 contains three variables 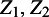 , and 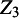 . Further, assume that the count variable 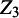 is sporadically and systematically missing in *m* and 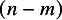 studies and 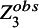 and 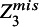 are the observed and missing part of 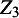 respectively. For 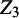 , we define the following Poisson mixed-effects model as the imputation model: 

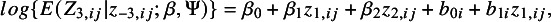

where the link function is the natural logarithm, and the model parameters are defined as in model 2.2. Assuming the parameters are a priori independent (i.e., 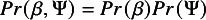 ) and specifying the standard prior distributions 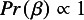 and 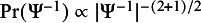 , the imputation procedure consists of the following steps:

1. Obtain the restricted maximum likelihood estimates 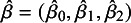 and 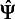 using 
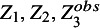
 .
2. Obtain the random effects 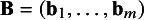 , where 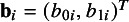 and calculate 
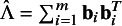
 .
3. Obtain a random draw 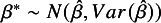 .
4. Obtain a random draw 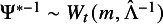 where *W* is a Wishart distribution with *m* degrees of freedom and a 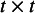 scale matrix parameter 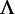 . (here 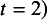

5. For each study *i*, 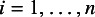

  - If 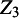 is sporadically missing, draw 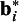 from the developed accept–reject sampling algorithm.
  - If 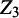 is systematically missing, draw 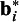 from 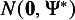 .
6. Impute 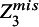 from the Poisson mixed-effects model 

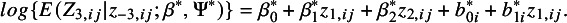

It should be noted that the scale matrix parameter 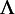 is calculated from studies for which 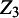 is sporadically missing and studies with systematically missing do not contribute, and consequently this may underestimate 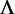 . Further, the above procedure can easily be modified for imputation of other types of categorical variables.

## 3 Simulations

A set of simulation studies was considered to assess the performance of the proposed multilevel imputation methodology by varying the between-study heterogeneity, the size of studies, the proportion of systematically missing data, the missing data mechanism, and the type of (incomplete) variables.

### 3.1 Simulation design

We began by generating a triple 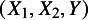 where 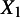 is a binary variable, 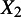 is a count variable, and *Y* is the outcome of interest (continuous or binary). Covariates 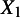 and 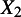 were generated from a joint mixed-effects model 

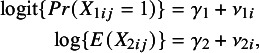

where 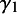 and 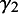 are the fixed-effects parameters and the random-effects 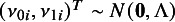 with 

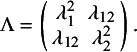

The continuous outcome variable *Y* was subsequently generated from the linear mixed-effects model 
(3.1)

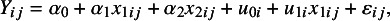

where 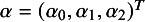 represents the regression coefficients of interest (i.e., the fixed-effects parameters), the residual errors 
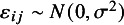
 , and the random effects 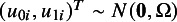 with 

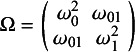

representing the random-effects parameters. For the binary outcome *Y*, we similarly defined the generalized linear mixed-effects model 
(3.2)

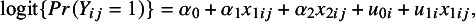

with the same assumption for 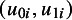 .

The parameter values were chosen to mimic the structure of an IPD-MA investigating potential factors associated with smoking in patients with CKD. Further details on the data are provided in the Application section. We chose alcohol consumption status and the number of comorbidities (e.g., hypertension, obesity, etc.) to motivate the distribution of the binary and count variables 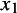 and 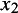 , respectively. For the outcome variable *y*, smoking status was used to motivate the parameters of the analysis model 3.2 (and 3.1). We considered three levels of between-study heterogeneity—weak, moderate, and strong—by varying elements of the covariance matrix 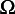 . Details about the parameter values used for the simulation study are provided in the Appendix.

For each simulated dataset, we introduced systematically and sporadically missing values in variables 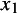 and 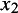 . These variables were independently systematically missing with probability 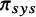 under the MCAR assumption, which is more plausible in IPD settings. We considered two proportions of systematically missing data: 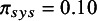 and 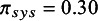 , resulting in approximately 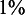 and 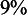 joint systematically missing data in both 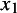 and 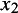 . In addition, for studies where a variable was not systematically missing, sporadically missing values were created independently for each variable under the MCAR and MAR assumptions. For the MAR scenarios, the probability that a data value is missing depended on the outcome variable (which was fully observed). Specifically, we used the following models to create sporadically missing data in 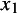 and 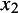 , respectively: 

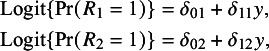

where 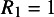 ( 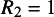 ) if 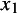 ( 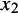 ) is observed and 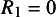 ( 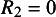 ) if 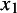 ( 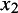 ) is sporadically missing. Under MCAR, 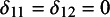 , and we fixed the proportion of sporadically missing data to 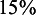 in each of 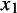 and 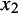 . Under MAR, we set 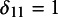 and 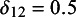 and adjusted the intercept values accordingly. For example, setting 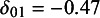 resulted in about 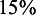 sporadically missing data in 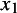 when the outcome variable *y* was continuous.

Finally, we considered five study sizes 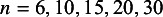 with a fixed total sample size of 3,000 participants, resulting in 500 subjects 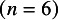 , 300 subjects 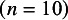 , 200 subjects 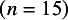 , 150 subjects 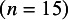 , and 100 subjects 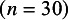 per study. This led to a total of 120 simulation scenarios (2 types of outcomes 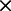 3 levels of between-study heterogeneity 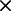 5 study sizes 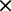 2 proportions of systematically missing data 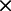 2 types of missing data mechanism), each of which was replicated 1000 times.

### 3.2 Methods and performance criteria

The simulation study evaluates five methods: the newly developed MLMI, the two-stage MLMI (2STG),^24^ the stratified imputation method (STI), complete case analysis (CCA), and the analysis of original data before the introduction of missing values (REF). The last method (REF) serves as a reference to assess the performance of the methods.

The STI method includes a study indicator as a categorical variable to the imputation model to account for the presence of clustering and uses standard imputation routines for non-clustered data, such as predictive mean matching, to generate imputations. However, for studies with systematically missing data, the intercept cannot be unidentified in the STI imputation method. To address this, the method uses the average of the observed study-intercept terms to obtain imputations for studies with systematically missing data. The CCA method, on the other hand, excludes subjects or studies with missing values from the analysis.

For all imputation methods (MLMI, 2STG, and STI), the conditional imputation models include all available covariates. In the MLMI and 2STG methods, the imputation models additionally include a random intercept and a random slope for the outcome variable *y*. Each incomplete dataset was imputed five times, with 10 cycles of the MICE algorithm used to obtain each imputed dataset. Afterward, each imputed dataset was fitted to the analysis model 3.1 or 3.2, and the results were pooled using Rubin’s rule. All analyses were conducted using the R packages 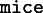 and 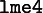 .

The primary parameters of interest are the fixed-effects parameters 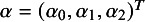 and the random-effects parameters 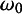 and 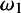 in model 3.1 or 3.2. For each method, we calculated the bias, root mean squared error (RMSE), model-based standard error (Model SE), empirical Monte Carlo standard error (Emp SE), and the coverage rate of the 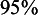 confidence interval (CR).

### 3.3 Results

This section presents the simulation study results for the binary outcome. The findings for the continuous outcome are included in the Supplementary Material.

#### 3.3.1 The binary outcome case

Tables 1–3 show the results for the fixed-effects and random-effects parameters with 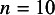 studies under MAR across the five different methods under weak, moderate, and strong between-study heterogeneity, respectively. Overall, all methods provided satisfactory estimates for the fixed-effects parameters 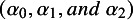 except for CCA where the bias for the estimate of 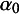 was moderate. Notably, both STI and 2STG exhibited a slight bias in the estimates of 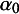 and 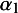 under strong between-study heterogeneity. Moreover, the CCA method had the largest RMSE, likely due to the proportion of missing data as it rose with the rate of systematically missing data. The RMSEs of the other methods were comparable.

**Table 1:** Estimates of the fixed- and random-effects parameters in the simulation study for the binary outcome with *n* = 10 studies and weak between-study heterogeneity

	Systematically missing									
	10%					30%				
	REF	CCA	STI	MLMI	2STG	REF	CCA	STI	MLMI	2STG
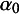 = 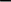 1.85										
Estimate	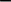 1.841	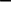 2.119	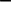 1.849	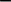 1.850	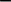 1.844	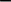 1.843	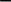 2.127	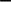 1.859	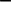 1.855	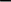 1.848
Bias	0.009	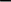 0.269	0.001	0.000	0.006	0.007	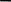 0.277	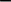 0.009	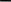 0.005	0.002
Model.SE	0.135	0.177	0.167	0.167	0.166	0.137	0.240	0.249	0.227	0.203
Emp.SE	0.141	0.178	0.163	0.162	0.160	0.140	0.246	0.205	0.190	0.181
CR%	91.4	66.8	93.3	94.2	94.3	91.3	77.4	96.6	95.8	95.7
RMSE	0.141	0.322	0.163	0.162	0.160	0.140	0.370	0.205	0.190	0.181
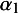 = 1.05										
Estimate	1.038	1.044	1.052	1.050	1.038	1.049	1.053	1.078	1.059	1.052
Bias	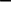 0.012	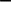 0.006	0.002	0.000	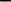 0.012	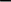 0.001	0.003	0.028	0.009	0.002
Model.SE	0.184	0.229	0.173	0.205	0.207	0.183	0.291	0.187	0.241	0.236
Emp.SE	0.190	0.230	0.213	0.213	0.214	0.191	0.305	0.243	0.249	0.241
CR%	91.9	92.9	88.4	90.9	91.0	91.9	90.2	84.3	90.3	91.1
RMSE	0.190	0.230	0.213	0.213	0.214	0.191	0.304	0.245	0.249	0.241
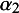 = 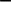 0.04										
Estimate	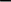 0.042	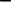 0.043	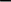 0.040	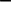 0.042	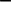 0.039	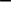 0.041	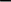 0.045	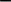 0.045	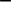 0.041	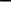 0.039
Bias	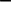 0.002	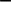 0.003	0.000	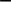 0.002	0.001	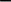 0.001	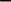 0.005	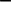 0.005	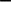 0.001	0.001
Model.SE	0.043	0.061	0.080	0.053	0.053	0.043	0.081	0.170	0.067	0.067
Emp.SE	0.042	0.061	0.057	0.050	0.051	0.043	0.078	0.093	0.062	0.062
CR%	95.7	95.0	98.0	95.3	94.6	95.3	96.2	99.0	94.4	94.9
RMSE	0.042	0.061	0.057	0.050	0.051	0.043	0.078	0.093	0.062	0.062
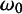 = 0.316										
Estimate	0.240	0.228	0.266	0.301	0.302	0.254	0.242	0.323	0.387	0.372
Bias	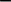 0.076	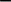 0.088	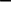 0.050	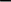 0.015	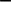 0.014	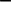 0.062	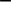 0.074	0.006	0.071	0.056
RMSE	0.176	0.202	0.155	0.150	0.145	0.167	0.229	0.146	0.234	0.157
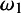 = 0.500										
Estimate	0.455	0.449	0.338	0.426	0.437	0.450	0.406	0.300	0.422	0.423
Bias	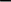 0.045	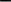 0.051	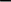 0.162	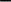 0.074	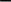 0.063	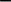 0.050	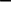 0.094	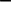 0.200	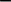 0.078	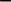 0.077
RMSE	0.162	0.203	0.198	0.188	0.180	0.157	0.254	0.228	0.221	0.205

*Note*: REF indicates the results that were obtained before missing data were introduced and can be viewed as a benchmark for comparing the performance of methods that are applied after missingness is introduced: complete case analysis (CCA), stratified multiple imputation (STI), multilevel multiple imputation (MLMI), and two-stage multilevel multiple imputation (2STG). The following values are given: mean of estimates (Estimate), bias (Bias), mean of standard error (Model SE), empirical standard error (Emp SE), the coverage rate of 95% confidence interval (CR), and the root of mean squared error (RMSE).

**Table 2:** Estimates of the fixed- and random-effects parameters in the simulation study for the binary outcome with n = 10 studies and moderate between-study heterogeneity

	Systematically missing									
	10%					30%				
	REF	CCA	STI	MLMI	2STG	REF	CCA	STI	MLMI	2STG
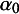 = 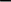 1.85										
Estimate	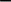 1.844	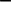 2.124	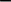 1.824	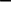 1.846	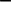 1.829	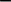 1.851	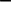 2.136	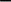 1.850	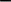 1.860	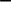 1.843
Bias	0.006	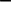 0.274	0.026	0.004	0.021	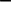 0.001	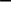 0.286	0.000	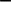 0.010	0.007
Model.SE	0.281	0.328	0.284	0.307	0.299	0.280	0.426	0.335	0.353	0.335
Emp.SE	0.302	0.356	0.320	0.322	0.316	0.297	0.453	0.349	0.348	0.334
CR%	90.5	81.7	89.7	91.5	91.1	90.7	83.9	91.4	93.0	93.2
RMSE	0.302	0.449	0.321	0.322	0.317	0.297	0.536	0.348	0.348	0.334
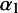 = 1.05										
Estimate	1.040	1.043	1.033	1.043	1.019	1.057	1.076	1.075	1.068	1.045
Bias	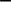 0.010	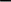 0.007	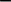 0.017	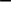 0.007	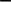 0.031	0.007	0.026	0.025	0.018	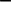 0.005
Model.SE	0.299	0.349	0.241	0.329	0.316	0.299	0.439	0.235	0.387	0.357
Emp.SE	0.311	0.376	0.337	0.348	0.336	0.315	0.473	0.378	0.391	0.377
CR%	89.6	89.8	81.2	88.9	88.6	91.8	88.6	75.5	89.6	89.0
RMSE	0.311	0.375	0.338	0.348	0.337	0.315	0.473	0.378	0.391	0.377
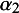 = 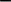 0.04										
Estimate	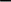 0.038	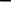 0.039	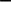 0.038	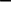 0.039	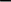 0.036	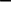 0.040	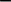 0.040	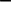 0.039	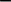 0.038	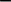 0.036
Bias	0.002	0.001	0.002	0.001	0.004	0.000	0.000	0.001	0.002	0.004
Model.SE	0.044	0.063	0.082	0.055	0.056	0.045	0.083	0.170	0.069	0.071
Emp.SE	0.044	0.064	0.060	0.054	0.054	0.045	0.082	0.092	0.064	0.063
CR%	95.8	94.8	97.7	94.5	95.1	93.9	94.4	99.3	93.5	95.4
RMSE	0.044	0.064	0.060	0.054	0.054	0.045	0.082	0.092	0.064	0.063
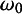 = 0.866										
Estimate	0.785	0.759	0.736	0.804	0.790	0.780	0.710	0.747	0.849	0.825
Bias	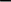 0.081	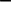 0.107	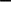 0.130	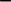 0.062	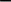 0.076	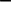 0.086	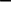 0.156	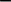 0.119	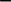 0.017	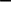 0.041
RMSE	0.241	0.312	0.250	0.232	0.230	0.248	0.407	0.244	0.235	0.221
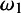 = 0.922										
Estimate	0.835	0.810	0.583	0.804	0.790	0.836	0.739	0.491	0.790	0.753
Bias	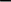 0.087	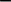 0.112	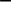 0.339	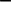 0.118	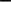 0.132	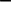 0.086	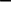 0.183	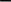 0.431	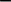 0.132	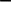 0.169
RMSE	0.261	0.325	0.388	0.307	0.299	0.251	0.411	0.468	0.359	0.337

*Note*: REF indicates the results that were obtained before missing data were introduced and can be viewed as a benchmark for comparing the performance of methods that are applied after missingness is introduced: complete case analysis (CCA), stratified multiple imputation (STI), multilevel multiple imputation (MLMI), and two-stage multilevel multiple imputation (2STG). The following values are given: mean of estimates (Estimate), bias (Bias), mean of standard error (Model SE), empirical standard error (Emp SE), the coverage rate of 95% confidence interval (CR), and the root of mean squared error (RMSE).

**Table 3:** Estimates of the fixed- and random-effects parameters in the simulation study for the binary outcome with n = 10 studies and strong between-study heterogeneity

	Systematically missing									
	10 %					30 %				
	REF	CCA	STI	MLMI	2STG	REF	CCA	STI	MLMI	2STG
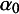 = 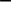 1.85										
Estimate	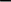 1.858	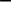 2.125	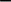 1.714	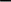 1.852	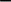 1.754	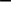 1.869	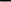 2.140	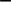 1.708	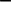 1.875	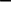 1.746
Bias	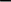 0.008	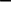 0.275	0.136	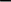 0.002	0.096	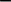 0.019	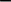 0.290	0.142	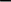 0.025	0.104
Model.SE	0.524	0.592	0.453	0.548	0.502	0.527	0.787	0.472	0.627	0.543
Emp.SE	0.540	0.616	0.526	0.563	0.511	0.551	0.843	0.587	0.634	0.569
CR%	90.9	87.7	86.2	91.9	91.8	91.9	85.5	85.1	93.4	92.0
RMSE	0.540	0.675	0.543	0.562	0.520	0.551	0.891	0.603	0.634	0.578
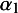 = 1.05										
Estimate	1.058	1.064	0.942	1.058	0.947	1.050	1.071	0.917	1.045	0.904
Bias	0.008	0.014	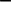 0.108	0.008	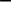 0.103	0.000	0.021	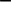 0.133	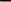 0.005	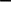 0.146
Model.SE	0.533	0.598	0.376	0.573	0.516	0.537	0.809	0.352	0.720	0.597
Emp.SE	0.550	0.642	0.562	0.594	0.549	0.560	0.852	0.642	0.702	0.634
CR%	91.4	89.4	78.6	91.8	89.9	91.1	86.8	68.9	89.8	86.7
RMSE	0.550	0.642	0.572	0.594	0.559	0.559	0.852	0.656	0.702	0.650
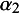 = 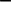 0.04										
Estimate	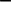 0.037	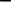 0.039	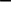 0.035	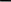 0.037	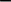 0.034	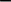 0.042	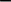 0.046	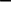 0.042	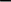 0.039	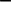 0.038
Bias	0.003	0.001	0.005	0.003	0.006	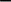 0.002	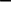 0.006	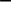 0.002	0.001	0.002
Model.SE	0.046	0.064	0.083	0.057	0.060	0.046	0.086	0.168	0.072	0.077
Emp.SE	0.044	0.063	0.058	0.052	0.053	0.045	0.083	0.093	0.063	0.066
CR%	95.8	96.3	98.3	97.5	96.1	95.8	95.4	98.9	94.9	95.9
RMSE	0.044	0.063	0.058	0.052	0.054	0.045	0.083	0.093	0.063	0.066
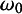 = 1.658										
Estimate	1.533	1.496	1.285	1.506	1.404	1.543	1.426	1.245	1.541	1.413
Bias	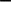 0.125	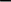 0.162	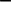 0.373	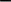 0.152	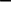 0.254	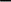 0.115	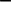 0.232	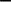 0.414	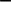 0.117	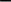 0.245
RMSE	0.442	0.520	0.507	0.436	0.442	0.432	0.719	0.523	0.438	0.429
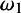 = 1.688										
Estimate	1.555	1.504	1.027	1.486	1.370	1.567	1.463	0.895	1.543	1.350
Bias	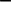 0.133	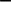 0.184	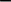 0.661	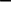 0.202	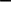 0.319	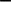 0.121	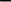 0.225	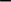 0.794	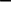 0.145	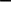 0.338
RMSE	0.442	0.514	0.712	0.480	0.517	0.437	0.737	0.838	0.587	0.587

*Note*: REF indicates the results that were obtained before missing data were introduced and can be viewed as a benchmark for comparing the performance of methods that are applied after missingness is introduced: complete case analysis (CCA), stratified multiple imputation (STI), multilevel multiple imputation (MLMI), and two-stage multilevel multiple imputation (2STG). The following values are given: mean of estimates (Estimate), bias (Bias), mean of standard error (Model SE), empirical standard error (Emp SE), the coverage rate of 95% confidence interval (CR), and the root of mean squared error (RMSE).

The performance of methods varied with respect to confidence interval coverage. For the coefficient 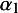 of the binary covariate, all methods were close to the nominal level since the variance of 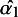 was generally underestimated, even for the reference method (REF). Nevertheless, the STI method had the lowest coverage rate, dropping below 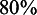 under strong between-study heterogeneity. Interestingly, STI resulted in coverage rates that exceeded the nominal level for the coefficient 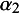 . These are likely due to improper modeling of the multilevel data structure during imputation. The other methods achieved the nominal 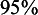 level for the coefficient 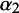 of the count variable. Despite not underestimating the variance of 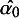 , the CCA method led to severe undercoverage for 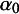 .

For the random-effects parameters 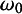 and 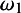 , all methods, including the reference, yielded downwardly biased estimates, likely due to the shrinkage effect in random-effects models and the limited number of studies, and the bias increased with the degree of between-study heterogeneity. For 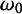 , the largest bias was observed with CCA under weak and moderate between-study heterogeneity, whereas it occurred with STI under strong between-study heterogeneity. For 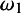 , the largest bias was consistently observed with STI across all between-study heterogeneity scenarios. In contrast, the MLMI and 2STG methods produced estimates close to the reference method, with 2STG exhibiting a slightly larger bias under strong between-study heterogeneity. When comparing RMSE, CCA, and STI had the highest values for 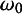 , and 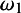 , respectively, followed by the MLMIs, where RMSE increased as the proportion of systematically missing data grew.

**Figure 1:**
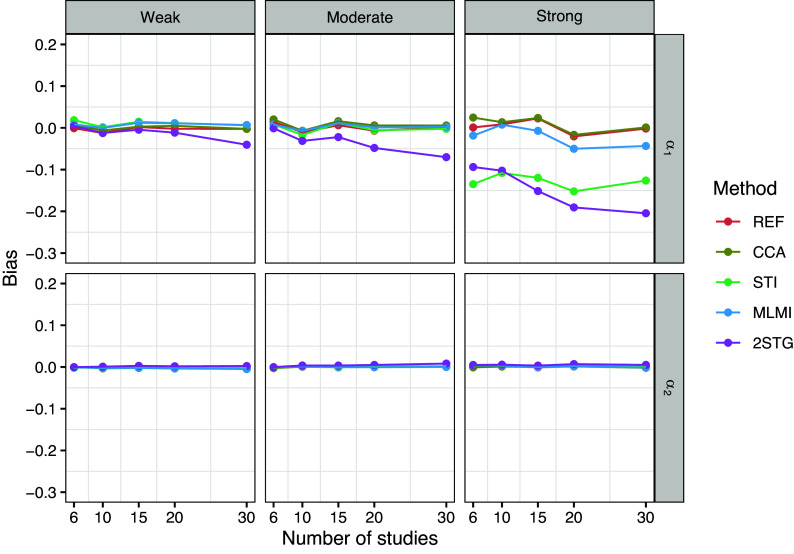
Bias of the fixed-effects estimates with 10% systematically missingness. Methods include reference (REF-before introducing missing data), complete case analysis (CCA), stratified multiple imputation (STI), multilevel multiple imputation (MLMI), and two-stage multilevel multiple imputation (2STG).

**Figure 2:**
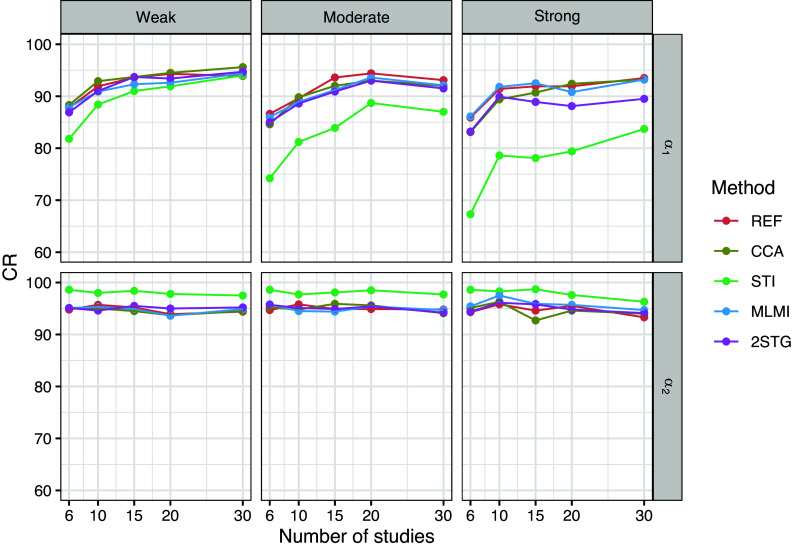
Coverage rate of the 95% confidence interval for the fixed-effects parameters with 10% systematically missingness. Methods include reference (REF-before introducing missing data), complete case analysis (CCA), stratified multiple imputation (STI), multilevel multiple imputation (MLMI), and two-stage multilevel multiple imputation (2STG).

**Figure 3:**
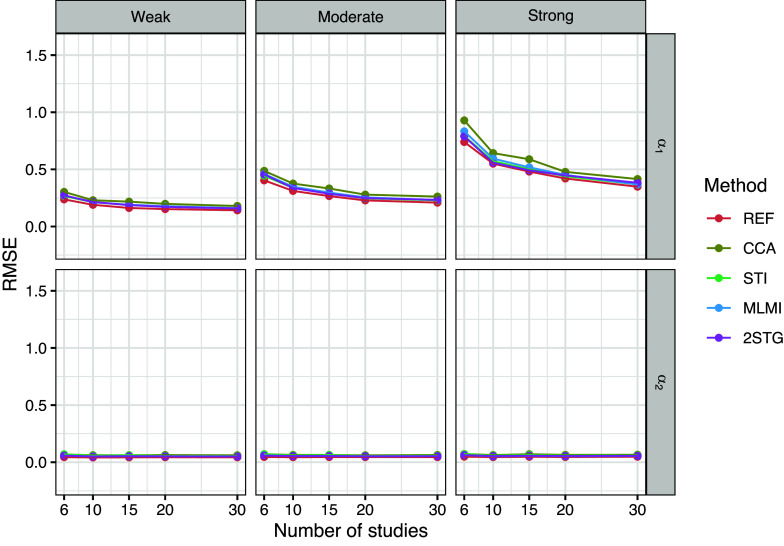
Root mean squared error (RMSE) of the fixed-effects estimates with 10% systematically missingness. Methods include reference (REF-before introducing missing data), complete case analysis (CCA), stratified multiple imputation (STI), multilevel multiple imputation (MLMI), and two-stage multilevel multiple imputation (2STG).

**Figure 4:**
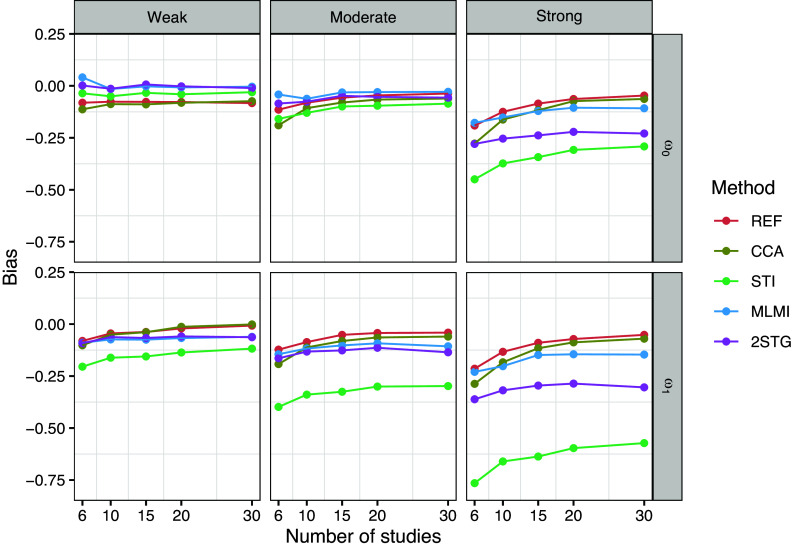
Bias of the random-effects estimates with 10% systematically missingness. Methods include reference (REF-before introducing missing data), complete case analysis (CCA), stratified multiple imputation (STI), multilevel multiple imputation (MLMI), and two-stage multilevel multiple imputation (2STG).

**Figure 5:**
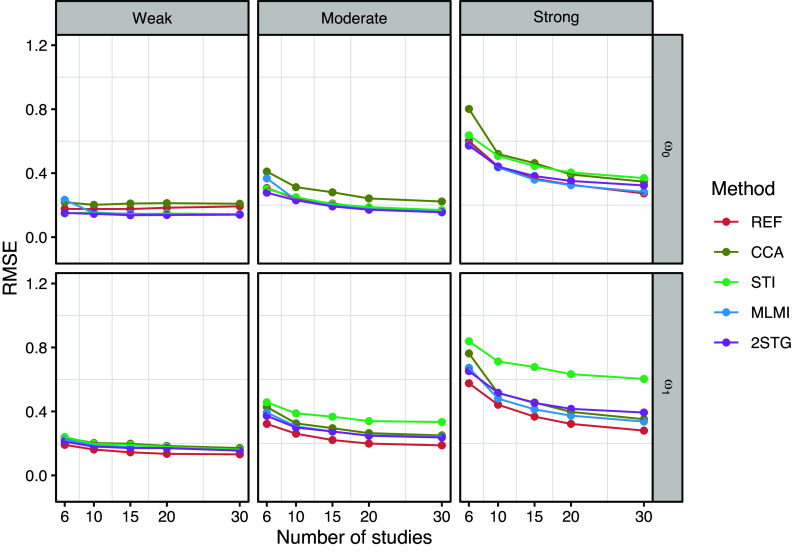
Root mean squared error (RMSE) of the random-effects estimates with 10% systematically missingness. Methods include reference (REF-before introducing missing data), complete case analysis (CCA), stratified multiple imputation (STI), multilevel multiple imputation (MLMI), and two-stage multilevel multiple imputation (2STG).

To evaluate the performance of the methods across different study sizes ( 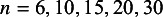 ), Figures 1–3 present the results for the fixed-effects parameters when the rate of systematically missing data was 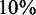 , and the missing data mechanism was MAR (for sporadically missing data). In Figures 4 and 5, the focus is on the random-effects parameters 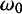 and 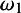 .

Figure 1 illustrates the bias in estimates of 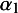 and 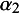 for the analysis model 3.2. Under weak and moderate between-study heterogeneity, all methods resulted in negligible bias for the coefficient 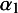 of the binary covariate, except for 2STG. Surprisingly, the 2STG method tended to underestimate 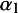 , with the bias gradually increasing as study size grew. Under strong between-study heterogeneity, both the 2STG and STI methods tended to underestimate 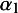 across different study sizes, while the MLMI method showed only a negligible bias in this scenario. For the coefficient 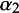 of the count covariate, on the other hand, bias remained trivial across all methods and study sizes.

Figure 2 presents the coverage rates of the 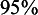 confidence intervals for the fixed-effects parameters 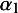 and 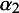 . STI exhibited significant coverage issues for the coefficient 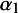 of the binary covariate, especially under moderate and strong between-study heterogeneity, where coverage dropped below 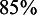 . Additionally, STI led to overcoverage for the coefficient 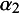 of the count covariate due to inflated standard errors across different study sizes and between-study heterogeneity scenarios. Conversely, the other methods performed reasonably well, maintaining coverage rates close to the nominal 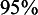 level (except for 2STG, which showed slightly lower coverage rates under strong between-study heterogeneity).

Figure 3 displays the RMSE of 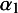 and 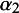 across different methods and study sizes. As expected, CCA showed the highest error, particularly for the coefficient 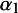 of the binary covariate with strong between-study heterogeneity. STI, 2STG, and MLMI had comparable RMSE values, though marginally higher than REF. We also noticed that RMSE of 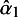 increased with the degree of between-study heterogeneity across all methods.

Figure 4 illustrates the bias in estimating the random-effects parameters 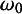 and 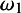 . As discussed earlier, all methods exhibited marginal underestimation of random-effects parameters. Most notably, STI suffered from a large bias, particularly when estimating the random-effect parameter 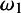 or when the between-study heterogeneity was strong. The multilevel imputation methods (MIML and 2STG) produced comparable estimates of the random-effects parameters and were generally in line with the reference method under weak and moderate between-study heterogeneity. However, under strong between-study heterogeneity, 2STG exhibited a noticeable bias, followed by MLMI, though the bias from MLMI remained trivial. Finally, CCA showed the least bias across different scenarios, although it increased with smaller study sizes.

Figure 5 shows the RMSE for the estimates of random-effects parameters 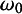 and 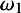 . For all methods, RMSE generally decreased as the number of studies increased but increased with greater between-study heterogeneity. CCA and STI consistently exhibited higher RMSE for 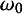 and 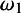 , respectively. In contrast, MLMI and 2STG tended to have lower RMSE, closely aligning with the reference method.

**Figure A1:**
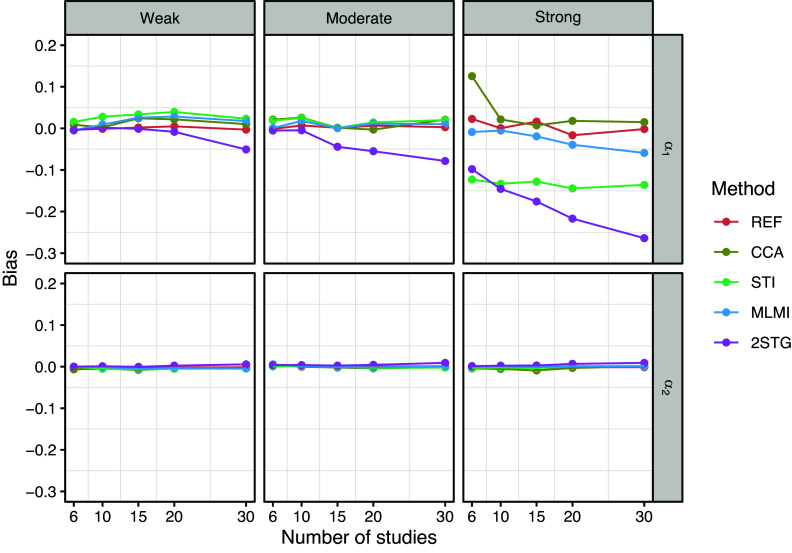
Bias of the fixed-effects estimates with 30% systematically missingness. Methods include reference (REF-before introducing missing data), complete case analysis (CCA), stratified multiple imputation (STI), multilevel multiple imputation (MLMI), and two-stage multilevel multiple imputation (2STG).

**Figure A2:**
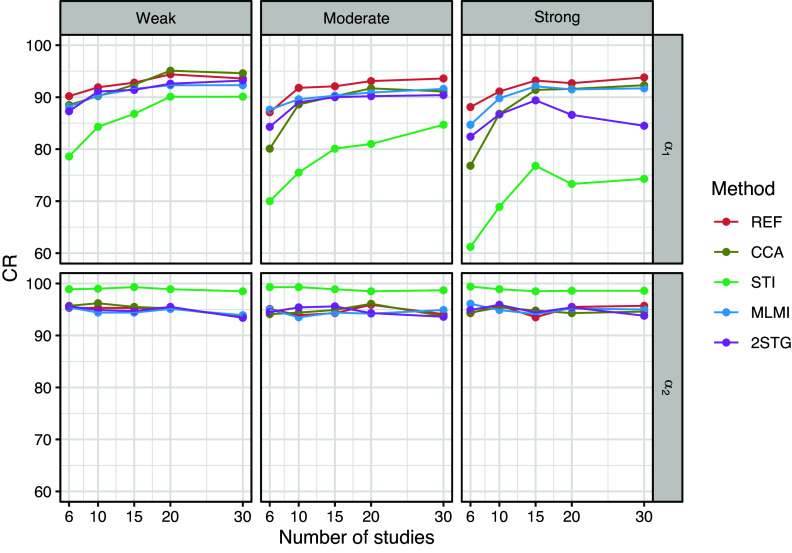
Coverage rate of the 95% confidence interval for the fixed-effects parameters with 30% systematically missingness. Methods include reference (REF-before introducing missing data), complete case analysis (CCA), stratified multiple imputation (STI), multilevel multiple imputation (MLMI), and two-stage multilevel multiple imputation (2STG).

**Figure A3:**
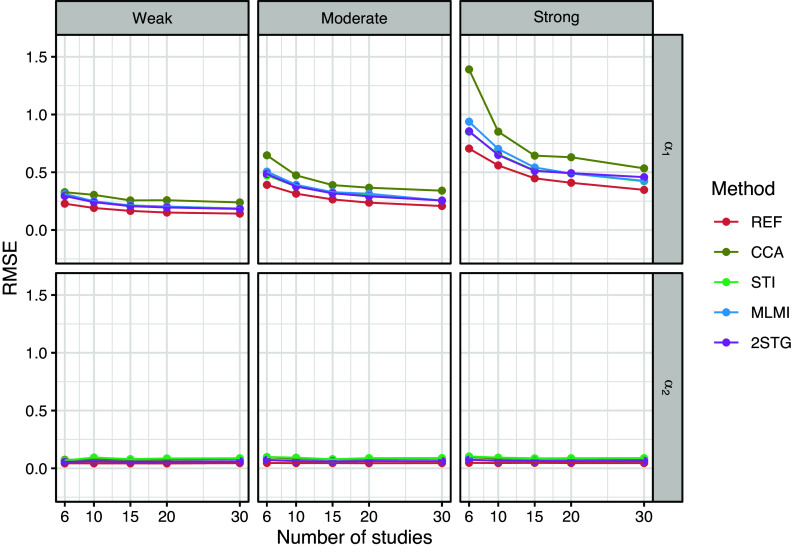
Root mean squared error (RMSE) of the fixed-effects estimates with 30% systematically missingness. Methods include reference (REF-before introducing missing data), complete case analysis (CCA), stratified multiple imputation (STI), multilevel multiple imputation (MLMI), and two-stage multilevel multiple imputation (2STG).

The findings for the binary outcome with 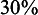 systematically missing data are presented in Figures A.1–A.5 in the Appendix. Although these results generally align with those in Figures 1–5, differences in model performance are more pronounced in Figures A.1–A.5 due to the higher rate of systematically missing data. Most notably, STI exhibited a substantial decline in coverage rates for the coefficient 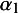 of the binary covariate, while it increased sharply for the coefficients 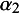 of the count covariate. Additionally, 2STG showed a gradual decline in coverage rates for 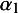 under strong between-study heterogeneity (Figure A2). Lastly, CCA showed a modest increase in RMSE of 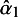 (Figure A3).

**Figure A4:**
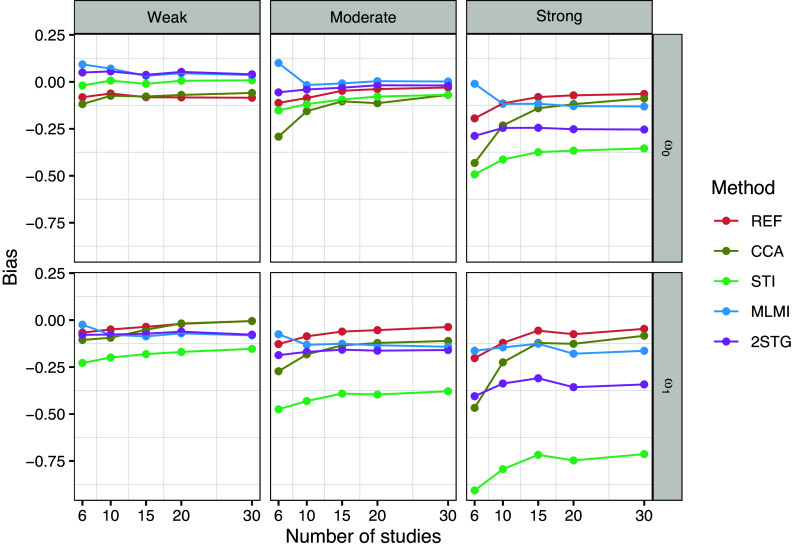
Bias of the random-effects estimates with 30% systematically missingness. Methods include reference (REF-before introducing missing data), complete case analysis (CCA), stratified multiple imputation (STI), multilevel multiple imputation (MLMI), and two-stage multilevel multiple imputation (2STG).

It is also worth noting that the performance of methods changed slightly when the number of studies was small ( 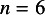 ) and the rate of systematically missing data was 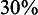 . Specifically, CCA exhibited a rapid decline in coverage rate for 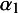 , dropping below 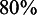 , whereas the decline was less pronounced for the other methods, including REF (Figure A2). Moreover, CCA produced downward-biased estimates for the random-effects parameters 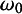 and 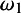 , while MLMI exhibited an upward bias only for 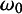 (Figure A4). This may be due to the limited number of studies available, which could affect imputation precision for MLMI and reduce analysis accuracy for CCA.

Finally, we did not report the results for MCAR cases (for sporadically missing data), as the methods showed no substantial performance differences across scenarios.

#### 3.3.2 The continuous outcome case

The results for the continuous outcome are presented in the Supplementary Material. Specifically, Tables S1–S3 in the Supplementary Material provide the results for the fixed-effects and random-effects parameters with 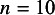 studies. Figures S1–S10 in the Supplementary Material illustrate the findings for 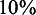 and 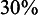 systematically missing data, respectively.

Overall, the performance of the methods remained consistent with that observed in the binary case. However, CCA performed poorly. Specifically, it exhibited the largest bias for the coefficient 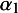 of the binary covariate across most scenarios, while 2STG had the second largest bias, but only when study size was large and between-study heterogeneity was strong (Figures S1 and S6 in the Supplementary Material). The bias with the other methods was fairly trivial. Furthermore, both CCA and STI showed coverage issues for 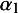 , whereas the other methods achieved improved coverage rates (Figures S2 and S7 in the Supplementary Material). For the coefficient 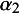 of the count covariate, all methods performed well in terms of bias and coverage rates, similar to the binary case.

For the random-effects parameters 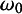 and 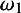 , CCA and STI showed biased estimates with CCA showing the largest bias for 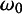 and STI for 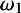 (Figures S4 and S9 in the Supplementary Material). MLMI and 2STG performed comparably, exhibiting less bias across different scenarios. Nevertheless, 2STG tended to show greater bias for 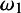 as study size and between-study heterogeneity increased.

As in the binary case, the methods showed no substantial performance differences across scenarios under the MCAR mechanism, so the results are not presented.

#### 3.3.3 Conclusion

In summary, the findings indicate that naïve methods like CCA and STI have major drawbacks. Specifically, CCA struggles with the reduced number of studies, leading to increased errors and potentially biased estimates, and lower coverage rates, particularly when the proportion of systematically missing data increases. Similarly, the STI method showed bias and coverage issues in many scenarios due to inadequate modeling of the multilevel structure. In contrast, the multilevel imputation methods (MLMI and 2STG), which appropriately account for the multilevel structure, offer more reliable inferences, with smaller bias and confidence intervals closer to the nominal level. Nonetheless, 2STG exhibited slight underestimation in some scenarios, possibly due to treating categorical variables with sporadically missing values as systematically missing and imputing them accordingly. When the study size was small ( 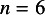 ), the performance of all methods generally declined, highlighting their limitations in settings with limited data.

## 4 Application

To demonstrate the approaches, we considered an IPD meta-analysis of 15 studies involving 4,774 patients with CKD stages III–V, defined by an estimated glomerular filtration rate below 60. The anonymized individual participant datasets for these studies were retrieved from the PLOS ONE website.

In this purely methodological exercise, we aimed to explore whether alcohol consumption and the number of existing health conditions such as hypertension and diabetes are associated with smoking status in this specific population. Focusing on the analysis model 3.2, we considered smoking status as the outcome *y* and alcohol consumption (yes vs no) and the number of comorbidities (count) as the covariates 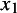 and 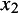 , respectively. The number of comorbidities was defined as the sum of the presence of hypertension, diabetes, obesity, and cardiovascular disease. While the analysis model was restricted to these variables, the imputation models also included additional covariates—age and sex—to make the MAR assumption more plausible.^29^ Table 4 summarizes the percentage of systematically and sporadically missing data for each variable included in this case study.

**Table 4:** Percentage of missing data by variable and study in the empirical example

Study	Size	Smoke	Gender	Age	Alcohol	Comorbidities
1	832	1	0	0	1	0
2	533	0	0	0	26	0
3	88	0	0	0	0	0
4	1,144	0	0	0	100	0
5	551	0	0	0	100	0
6	578	0	0	0	100	0
7	116	0	0	0	100	0
8	44	39	0	0	55	0
9	73	0	0	0	5	0
10	55	0	0	0	100	0
11	27	0	0	0	100	0
12	405	0	0	0	100	0
13	224	0	0	0	0	0
14	59	0	0	0	100	100
15	45	0	0	0	100	0

We employed four methods to investigate the strength of association and the degree of between-study heterogeneity for the covariates in model 3.2. First, we performed the CCA, excluding studies with systematically missing data and patients with sporadically missing data. This resulted in a subset of 1,615 patients across six studies.

Second, we applied the stratified MI method (STI), which accounts for heterogeneous intercepts by including study indicators in the imputation model. For STI, binary and count variables were imputed using the *logreg* and *pmm* imputation procedures in the R package 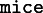 , respectively.

Finally, we applied the newly developed MLMI to account for heterogeneity across studies by incorporating joint random effects into the imputation models. Specifically, the imputation models for alcohol consumption (binary) and the number of comorbidities (count) included random effects on the intercept and the outcome variable smoking status, while the other covariates were treated as fixed effects. In addition, the two-stage MLMI (2STG) was included for comparison. For 2STG, the imputation models were identical to those used in MLMI.

For all imputation approaches (STI, 2STG, and MLMI), we allowed 20 iterations of the MICE algorithm to enhance convergence. Following White et al.,^15^ the number of imputed datasets should be at least as large as the percentage of missing data. Since approximately 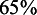 of the data were missing in this study, we generated 100 imputed datasets from the original data.

Table 5 presents the estimates of the fixed-effects coefficients, their standard errors, and the between-study heterogeneity estimates (i.e., the random-effects parameters 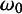 and 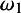 ) for this case study. Overall, the results align with the findings from the simulation study.

**Table 5:** Estimates of the fixed- and random-effects parameters in the empirical example

	Fixed effects						Random effects	
	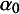		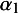		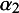		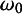	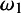
	Est	se	Est	se	Est	se	Est	Est
CCA	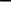 1.794	0.572	0.913	0.477	0.102	0.085	1.217	0.677
STI	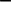 1.289	0.315	0.632	0.265	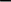 0.014	0.045	1.129	0.543
MLMI	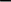 1.322	0.353	0.677	0.403	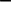 0.012	0.047	1.137	0.616
2STG	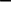 1.125	0.459	0.372	0.600	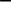 0.005	0.048	1.286	0.975

*Note*: Methods include complete case analysis (CCA), stratified multiple imputation (STI), multilevel multiple imputation (MLMI), and two-stage multilevel multiple imputation (2STG).

First, the estimates of the random-effects parameters indicate moderate between-study heterogeneity. The STI method tended to underestimate this heterogeneity, particularly for 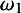 , whereas other methods produced comparable estimates. Surprisingly, 2STG overestimated 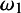 , with its estimate increasing sharply compared to CCA and MLMI.

Next, CCA produced fixed-effects coefficient estimates that were further from the null compared to those obtained using the MI methods. This suggests that the missing data mechanism might not be completely at random. The MI methods yielded similar estimates for the fixed-effects parameters, but the estimate of the regression slope for alcohol consumption ( 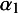 ) appeared to differ with 2STG compared to the other imputation methods. Although this pattern was also observed in the simulation study (middle panel of Figure A1 in the Appendix), the corresponding coefficient is not statistically significant at the 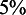 level, suggesting that differences in point estimates are unlikely to be meaningful.

As expected, CCA overestimated the standard errors of the fixed-effects parameters due to substantial data reduction. In contrast, the STI method underestimated these standard errors relative to the multilevel imputation approaches, likely due to improper handling of between-study heterogeneity.

In summary, the multilevel imputation methods (particularly MLMI), which properly account for between-study heterogeneity, may provide more accurate results than other alternatives.

## 5 Discussion

Meta-analysis of IPD should employ appropriate methodologies to deal with the simultaneous presence of systematically and sporadically missing data. Excluding incomplete studies or cases from the meta-analysis is undesirable, as full data potential is not optimally used, especially when the individual studies are too large or important to be excluded. This can lead to efficiency loss, reduced statistical power due to a smaller sample size, and potentially biased estimates.

Additionally, in the simulation study, CCA yielded conflicting results when comparing binary and continuous outcomes. Specifically, the estimates of the main parameters ( 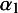 and 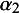 ) were unbiased for the binary outcome but exhibited bias in the continuous case. This is not surprising because CCA leads to biased regression slope estimates in linear regression when missingness in a covariate depends on the outcome variable *Y* (i.e., the MAR mechanism). In contrast, in logistic regression with CCA, the regression slope estimates remain unbiased when missingness depends on *Y* due to the symmetry property of the odds ratio (see Bartlett et al.^30^ and Carpenter and Kenward^31^).

Hence, imputation strategies become an attractive solution in meta-analysis of IPD. Naïve imputation approaches, such as stratified imputation, which do not fully account for variability within and across studies typically lead to biased parameter estimates, and hence wrong conclusions may be drawn.^18^ Multilevel (or hierarchical) imputation approaches are therefore a valuable alternative to preserve data integrity in IPD-MA and should be preferred in practice.

Building upon our previous work, we developed new multilevel imputation methodologies for clustered (or multilevel) data, which effectively handle the combined presence of systematically and sporadically missing values in dichotomous or count variables. This methodology utilizes a generalized linear mixed-effects model with random intercept terms and random slopes to complete the imputation task. The simulation and case study results demonstrate that the proposed multilevel imputation approach has desirable statistical properties in terms of bias and coverage rates and maintains appropriate levels of between-study heterogeneity. Furthermore, this methodology facilitates more complex post-imputation analyses by incorporating joint random effects during the imputation phase. Finally, the developed multilevel imputation routines are freely accessible via the popular R package 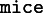 .

Within the MICE framework, missing values can be imputed per study, a process known as the two-stage imputation approach as opposed to the one-stage imputation approach (such as the proposed MLMI methodology) that imputes missing values within and across studies. The two-stage imputation approach does not impose any restrictions on sporadically missing data. However, it is no longer feasible to impute systematically missing data because the variable is entirely missing in certain studies, so no imputation can be generated for that variable within those studies.

Resche-Rigon and White^24^ proposed a variant of the two-stage imputation approach (the 2STG method in the simulation study), where a two-stage estimator is used to handle both types of missing data. Compared to our one-stage imputation approach, the two-stage imputation approach^24^ is computationally faster because estimation is performed in two steps. However, in our simulations, this approach produced mildly biased estimates for categorical covariates, particularly when the between-study heterogeneity was strong. A possible explanation is that it treats categorical variables with sporadically missing data as systematically missing, leading to inaccurate imputations. In addition, this method requires large studies to minimize the small-sample bias of the maximum likelihood estimator and to avoid separability issues, particularly with binary variables. Moreover, it is prone to overfitting when there are many covariates or high rates of missing data within each study. In contrast, the one-stage imputation approach is highly relevant and useful for IPD sets even with relatively few studies, as it can prevent overfitting issues. Audigier et al.^19^ compared broadly properties of the one-stage and two-stage imputation approaches (together with other imputation methods) through an extensive simulation study.

One limitation of the one-stage imputation approaches within the MICE framework is the compatibility issue inherited from the standard (non-hierarchical) FCS algorithm. The conditional multilevel imputation models may not be consistent with a well-defined joint multilevel model, meaning the imputation models could be generally misspecified.^24^ ^,^ ^32^ However, both papers showed via simulation studies that the impact of misspecified imputation models could be minor, particularly when the number of studies is large. Additionally, one-stage imputation approaches, such as MLMI, are computationally intensive and slow due to fitting several mixed-effects models during imputation. This is, however, less of an issue nowadays with fast processors and parallel computing options.

Multilevel imputation models often involve many random effects, which can cause convergence issues, particularly when the number of studies is limited. This can lead to inaccurate imputations, introducing bias in subsequent analyses. As suggested by Jolani et al.,^25^ simplifying the imputation model (e.g., by considering fewer or independent random effects) may help address this issue. Furthermore, in our simulation study, Rubin’s rule was applied to summarize the random-effects parameters (i.e., taking the arithmetic average of the estimated variances of the random effects from the imputed datasets). However, given the skewness of the distribution of these parameters, taking a simple mean might not be the optimal approach. Increasing the number of imputations and using the median of the estimated random parameters could be a better alternative, although further research is needed to systematically evaluate this proposal.

It is also worth mentioning that in IPD-MA, variables with systematically missing data are typically assumed to follow an MCAR or MAR mechanism. This assumption enables leveraging information from studies with observed data to impute plausible values for studies with systematically missing data. However, the possibility of an MNAR mechanism cannot be ruled out. A potential solution is to integrate the proposed multilevel imputation approach with the pattern-mixture approach and conduct a sensitivity analysis.

Although the development of the new imputation methodology was motivated by its usage in the meta-analysis of IPD, this methodology, by no means, is restricted to studies with both systematically and sporadically missing data. The proposed imputation methodology is general and can be directly applied to other hierarchical or multilevel settings, such as longitudinal studies where sporadically missing data are common.

While we focused on logistic and Poisson mixed-effects models in the current article, extending this methodology to other families of distributions, such as gamma or inverse Gaussian, would be relatively straightforward. Additionally, extending the methodology to handle other types of categorical variables (nominal and ordinal) with both systematically and sporadically missing data would be a valuable direction for future research. Another promising line of research is to broaden the methodology to handle treatment-covariate interactions (i.e., effect modifiers) in IPD-MA. Our current methodology does not account for effect modifiers during the imputation process, and addressing this challenge is not trivial. When a covariate has missing observations, its interaction with treatment will also be incomplete, complicating the imputation process.

## Supporting information

Jolani supplementary material Jolani supplementary material

## Figures and Tables

**Figure A5:**
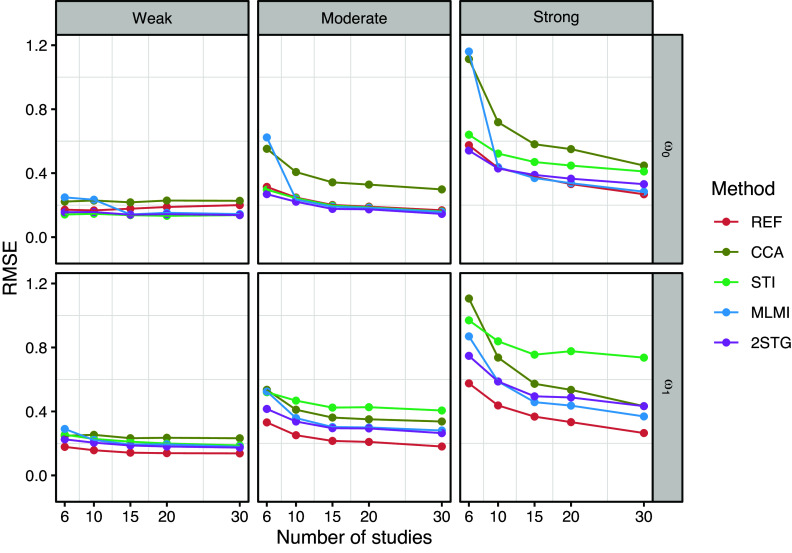
Root mean squared error (RMSE) of the random-effects estimates with 30% systematically missingness. Methods include reference (REF-before introducing missing data), complete case analysis (CCA), stratified multiple imputation (STI), multilevel multiple imputation (MLMI), and two-stage multilevel multiple imputation (2STG).

## Data Availability

R code to reproduce the simulation results is available on GitHub (https://github.com/shahabjolani/mlmi-categorical).

## Supplementary material

The supplementary material for this article can be found at https://doi.org/10.1017/rsm.2025.10017.

## A Setup values for simulation study

The parameters were

and

We considered three versions of between-study heterogeneity by varying the covariance matrix  :

- Strong between-study heterogeneity:
- Moderate between-study heterogeneity:
- Weak between-study heterogeneity:

For the continuous outcome scenarios, we set  .

## B Simulation results for the binary outcome with 30% systematically missingness

Figures A1–A5 present the findings of binary outcome when the rate of systematically missing data was 30%.
