# Supplementary material for: Hierarchical imputation of categorical variables in the presence of systematically and sporadically missing data
Source: Res Synth Methods. 2025 Jun 10;16(5):729–57. doi: 10.1017/rsm.2025.10017 (PMC12527547; doi:10.1017/rsm.2025.10017)
Supplement: Jolani supplementary material [file S1759287925100173sup001.pdf]

Hierarchical imputation of categorical variables in the presence of systematically and sporadically missing data

A Preprint

Shahab Jolani
Department of Methodology and Statistics
Care and Public Health Research Institute (CAPHRI), Maastricht University
Maastricht, 6229 HA, The Netherlands
s.jolani@maastrichtuniversity.nl

March 10, 2025

Abstract

1 Supplementary materials

1.1 Simulation results for the continuous outcome with $n = 10$

Table 1-3 show the results of the continuous outcome for the fixed-effects and random-effects parameters across the five different methods under weak, moderate and strong between-study heterogeneity.

Table 1: Estimates of the fixed- and random-effects parameters in the simulation study for the continuous outcome with n = 10 studies and weak between-study heterogeneity

	Systematically missing									
	10 %					30 %				
	REF	CCA	STI	MLMI	2STG	REF	CCA	STI	MLMI	2STG
α_0 **= -1.85**										
Estimate	-1.849	-2.017	-1.835	-1.838	-1.839	-1.851	-2.019	-1.847	-1.826	-1.831
Bias	0.001	-0.167	0.015	0.012	0.011	-0.001	-0.169	0.003	0.024	0.019
Model.SE	0.105	0.108	0.109	0.114	0.112	0.106	0.142	0.115	0.137	0.126
Emp.SE	0.105	0.115	0.110	0.110	0.109	0.107	0.144	0.127	0.124	0.121
CR	93.000	62.700	92.000	93.500	93.000	90.500	69.000	89.900	93.600	92.800
RMSE	0.105	0.203	0.111	0.111	0.109	0.107	0.222	0.127	0.127	0.122
α_1 **= 1.05**										
Estimate	1.045	0.910	1.030	1.034	1.032	1.054	0.917	1.040	1.025	1.029
Bias	-0.005	-0.140	-0.020	-0.016	-0.018	0.004	-0.133	-0.010	-0.025	-0.021
Model.SE	0.162	0.157	0.133	0.166	0.160	0.163	0.206	0.125	0.187	0.170
Emp.SE	0.166	0.163	0.172	0.172	0.171	0.159	0.209	0.181	0.187	0.183
CR	93.000	80.600	83.900	90.800	90.400	92.700	82.200	78.900	89.100	88.100
RMSE	0.166	0.214	0.173	0.173	0.172	0.159	0.247	0.181	0.189	0.184
α_2 **= -0.04**										
Estimate	-0.040	-0.036	-0.040	-0.040	-0.039	-0.040	-0.034	-0.039	-0.038	-0.039
Bias	0.000	0.004	0.000	0.000	0.001	0.000	0.006	0.001	0.002	0.001
Model.SE	0.018	0.022	0.024	0.023	0.023	0.017	0.029	0.035	0.029	0.031
Emp.SE	0.017	0.022	0.023	0.022	0.022	0.017	0.029	0.030	0.026	0.026
CR	95.400	94.800	95.300	95.500	95.400	96.000	94.000	93.800	94.300	95.900
RMSE	0.017	0.022	0.023	0.022	0.022	0.017	0.030	0.030	0.026	0.026
ω_0 **= 0.316**										
Estimate	0.301	0.268	0.303	0.308	0.308	0.305	0.263	0.307	0.336	0.324
Bias	-0.015	-0.048	-0.013	-0.008	-0.008	-0.011	-0.053	-0.009	0.020	0.007
RMSE	0.082	0.100	0.080	0.080	0.080	0.084	0.126	0.082	0.090	0.081
ω_1 **= 0.500**										
Estimate	0.482	0.408	0.383	0.463	0.457	0.487	0.394	0.343	0.455	0.434
Bias	-0.018	-0.092	-0.117	-0.037	-0.043	-0.013	-0.106	-0.157	-0.045	-0.066
RMSE	0.121	0.153	0.155	0.134	0.132	0.121	0.201	0.187	0.157	0.153

Note:
REF indicates the results that were obtained before missing data were introduced and can be viewed as a benchmark for comparing the performance of methods that are applied after missingness is introduced: complete case analysis (CCA), stratified multiple imputation (STI), multilevel multiple imputation (MLMI), and two-stage multilevel multiple imputation (2STG). The following values are given: mean of estimates (Estimate), bias (Bias), mean of standard error (Model SE), empirical standard error (Emp SE), the coverage rate of 95% confidence interval (CR), and the root of mean squared error (RMSE)

Table 2: Estimates of the fixed- and random-effects parameters in the simulation study for the continuous outcome with n = 10 studies and moderate between-study heterogeneity

	Systematically missing									
	10 %					30 %				
	REF	CCA	STI	MLMI	2STG	REF	CCA	STI	MLMI	2STG
**α_0 = -1.85**										
Estimate	-1.867	-2.057	-1.843	-1.855	-1.853	-1.857	-2.034	-1.846	-1.822	-1.828
Bias	-0.017	-0.207	0.007	-0.005	-0.003	-0.007	-0.184	0.004	0.028	0.022
Model.SE	0.274	0.275	0.264	0.278	0.275	0.274	0.356	0.259	0.294	0.280
Emp.SE	0.282	0.306	0.295	0.291	0.288	0.282	0.379	0.313	0.306	0.302
CR	92.000	81.800	90.600	91.600	91.500	90.900	82.100	85.900	91.600	91.100
RMSE	0.283	0.369	0.295	0.291	0.288	0.282	0.421	0.313	0.307	0.303
**α_1 = 1.05**										
Estimate	1.057	0.925	1.036	1.038	1.033	1.048	0.912	1.034	1.006	1.010
Bias	0.007	-0.125	-0.014	-0.012	-0.017	-0.002	-0.138	-0.016	-0.044	-0.040
Model.SE	0.297	0.290	0.235	0.303	0.288	0.294	0.370	0.210	0.337	0.290
Emp.SE	0.299	0.295	0.315	0.313	0.308	0.301	0.379	0.353	0.356	0.343
CR	91.200	87.400	80.500	90.800	89.700	90.200	86.500	70.000	87.500	84.100
RMSE	0.299	0.321	0.315	0.313	0.308	0.301	0.403	0.353	0.359	0.345
**α_2 = -0.04**										
Estimate	-0.040	-0.035	-0.039	-0.038	-0.037	-0.039	-0.036	-0.040	-0.038	-0.038
Bias	0.000	0.005	0.001	0.002	0.003	0.001	0.004	0.000	0.002	0.002
Model.SE	0.018	0.023	0.025	0.024	0.024	0.018	0.030	0.037	0.029	0.033
Emp.SE	0.018	0.022	0.024	0.022	0.022	0.017	0.029	0.030	0.025	0.027
CR	94.300	95.200	95.100	94.400	94.800	95.300	95.600	94.900	94.500	96.100
RMSE	0.018	0.022	0.024	0.022	0.022	0.017	0.029	0.030	0.025	0.027
**ω_0 = 0.866**										
Estimate	0.835	0.744	0.798	0.817	0.825	0.837	0.720	0.772	0.811	0.807
Bias	-0.031	-0.122	-0.068	-0.049	-0.041	-0.029	-0.146	-0.094	-0.055	-0.059
RMSE	0.205	0.238	0.206	0.204	0.205	0.200	0.311	0.207	0.194	0.198
**ω_1 = 0.922**										
Estimate	0.904	0.781	0.703	0.858	0.848	0.895	0.748	0.614	0.831	0.778
Bias	-0.018	-0.141	-0.219	-0.064	-0.074	-0.027	-0.174	-0.308	-0.091	-0.144
RMSE	0.221	0.261	0.282	0.227	0.232	0.218	0.334	0.353	0.261	0.270

Note:
REF indicates the results that were obtained before missing data were introduced and can be viewed as a benchmark for comparing the performance of methods that are applied after missingness is introduced: complete case analysis (CCA), stratified multiple imputation (STI), multilevel multiple imputation (MLMI), and two-stage multilevel multiple imputation (2STG). The following values are given: mean of estimates (Estimate), bias (Bias), mean of standard error (Model SE), empirical standard error (Emp SE), the coverage rate of 95% confidence interval (CR), and the root of mean squared error (RMSE)

Table 3: Estimates of the fixed- and random-effects parameters in the simulation study for the continuous outcome with n = 10 studies and strong between-study heterogeneity

	Systematically missing									
	10 %					30 %				
	REF	CCA	STI	MLMI	2STG	REF	CCA	STI	MLMI	2STG
α_0 **= -1.85**										
Estimate	-1.831	-2.058	-1.808	-1.825	-1.797	-1.851	-2.082	-1.854	-1.824	-1.784
Bias	0.019	-0.208	0.042	0.025	0.053	-0.001	-0.232	-0.004	0.026	0.066
Model.SE	0.525	0.523	0.477	0.518	0.510	0.536	0.693	0.466	0.541	0.509
Emp.SE	0.510	0.544	0.526	0.524	0.510	0.499	0.692	0.549	0.537	0.526
CR	92.700	88.600	89.700	92.000	92.000	93.400	87.300	87.200	92.200	91.600
RMSE	0.510	0.583	0.527	0.524	0.512	0.499	0.729	0.549	0.538	0.530
α_1 **= 1.05**										
Estimate	1.016	0.916	1.046	1.003	0.973	1.035	0.934	1.064	0.972	0.926
Bias	-0.034	-0.134	-0.004	-0.047	-0.077	-0.015	-0.116	0.014	-0.078	-0.124
Model.SE	0.535	0.525	0.400	0.540	0.499	0.541	0.699	0.369	0.609	0.515
Emp.SE	0.517	0.502	0.530	0.539	0.515	0.521	0.676	0.597	0.593	0.576
CR	93.400	92.100	83.000	92.200	90.800	93.000	86.900	72.000	90.200	87.000
RMSE	0.518	0.519	0.530	0.540	0.520	0.521	0.686	0.597	0.598	0.589
α_2 **= -0.04**										
Estimate	-0.040	-0.037	-0.039	-0.037	-0.036	-0.040	-0.035	-0.038	-0.034	-0.035
Bias	0.000	0.003	0.001	0.003	0.004	0.000	0.005	0.002	0.006	0.005
Model.SE	0.018	0.023	0.028	0.025	0.027	0.018	0.030	0.040	0.032	0.037
Emp.SE	0.018	0.023	0.025	0.022	0.024	0.018	0.030	0.033	0.027	0.029
CR	94.300	94.900	95.500	96.400	94.900	95.100	94.400	95.100	93.600	95.800
RMSE	0.018	0.023	0.025	0.022	0.024	0.018	0.030	0.033	0.027	0.029
ω_0 **= 1.658**										
Estimate	1.613	1.430	1.458	1.532	1.546	1.641	1.419	1.411	1.504	1.492
Bias	-0.045	-0.228	-0.200	-0.126	-0.113	-0.017	-0.239	-0.247	-0.154	-0.167
RMSE	0.383	0.449	0.397	0.381	0.374	0.406	0.575	0.428	0.403	0.395
ω_1 **= 1.688**										
Estimate	1.646	1.438	1.217	1.521	1.483	1.658	1.419	1.100	1.522	1.401
Bias	-0.042	-0.250	-0.471	-0.167	-0.205	-0.030	-0.269	-0.589	-0.166	-0.287
RMSE	0.380	0.449	0.554	0.397	0.398	0.405	0.609	0.668	0.445	0.470

Note:
REF indicates the results that were obtained before missing data were introduced and can be viewed as a benchmark for comparing the performance of methods that are applied after missingness is introduced: complete case analysis (CCA), stratified multiple imputation (STI), multilevel multiple imputation (MLMI), and two-stage multilevel multiple imputation (2STG). The following values are given: mean of estimates (Estimate), bias (Bias), mean of standard error (Model SE), empirical standard error (Emp SE), the coverage rate of 95% confidence interval (CR), and the root of mean squared error (RMSE)

1.2 Simulation results for the continuous outcome with 10% systematically missingness

The performance of the methods is shown in Figures 1-3 for the fixed-effects parameters and in Figures 4-5 for the random-effects parameters with 10% systematically missing data.

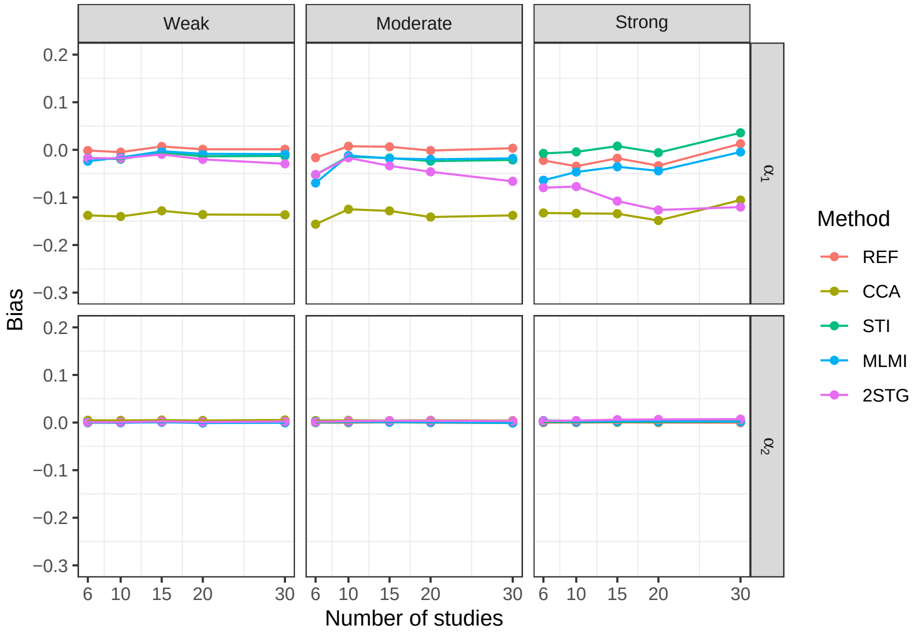

Figure 1: Bias of the fixed-effects estimates with 10% systematiclly missingness. Methods include reference (REF - before introducing missing data), complete case analysis (CCA), stratified multiple imputation (STI), multilevel multiple imputation (MLMI), and two-stage multilevel multiple imputation (2STG).

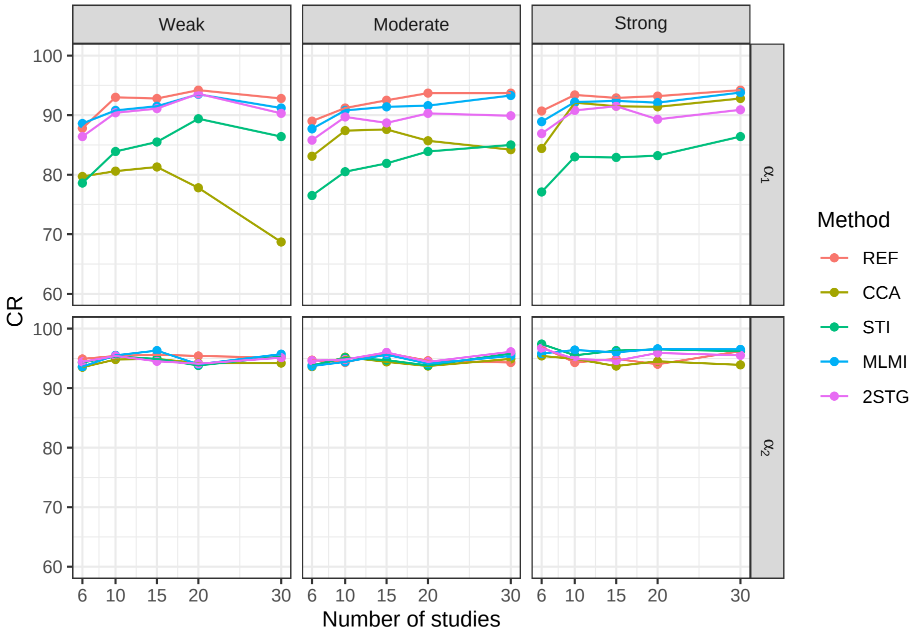

Figure 2: Coverage rate of the 95% confidence interval for the fixed-effects parameters with 10% systematiclly missingness. Methods include reference (REF - before introducing missing data), complete case analysis (CCA), stratified multiple imputation (STI), multilevel multiple imputation (MLMI), and two-stage multilevel multiple imputation (2STG).

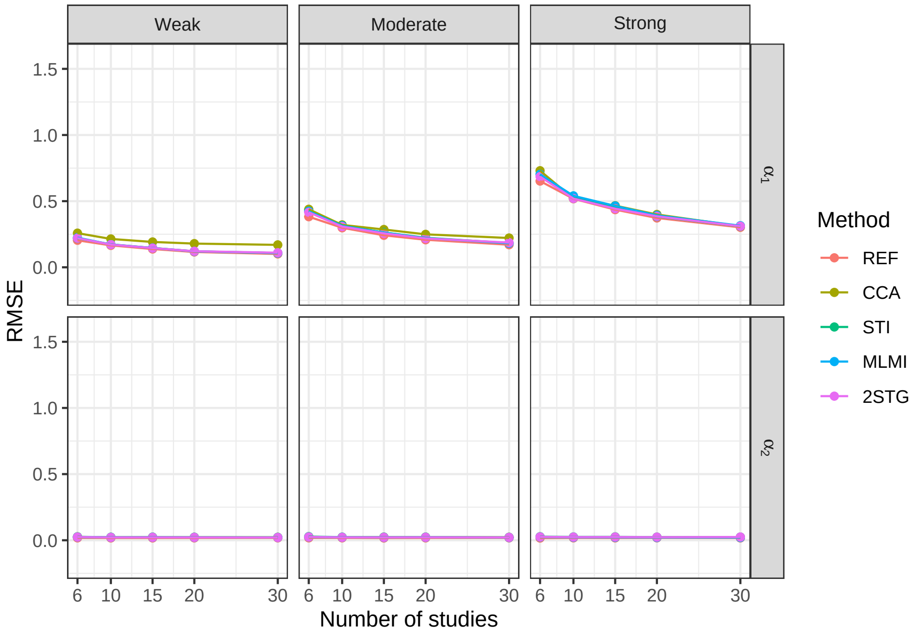

Figure 3: Root mean squared error (RMSE) of the fixed-effects estimates with 10% systematiclly missingness. Methods include reference (REF - before introducing missing data), complete case analysis (CCA), stratified multiple imputation (STI), multilevel multiple imputation (MLMI), and two-stage multilevel multiple imputation (2STG).

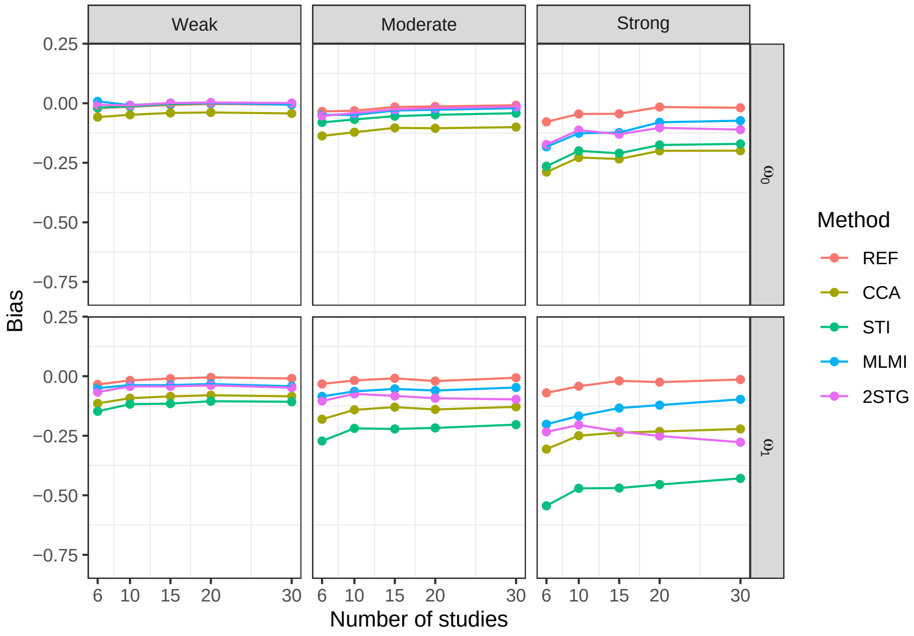

Figure 4: Bias of the random-effects estimates with 10% systematiclly missingness. Methods include reference (REF - before introducing missing data), complete case analysis (CCA), stratified multiple imputation (STI), multilevel multiple imputation (MLMI), and two-stage multilevel multiple imputation (2STG).

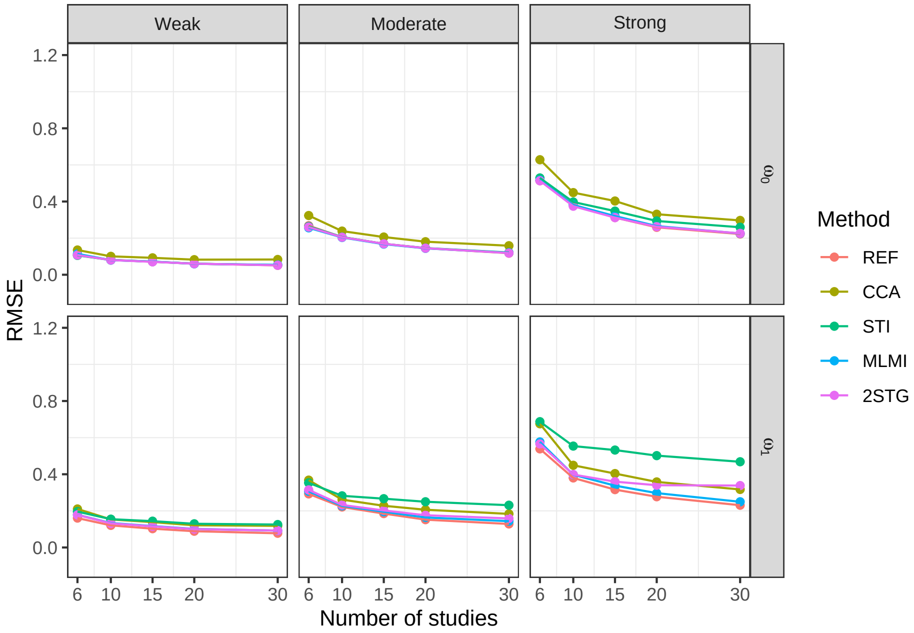

Figure 5: Root mean squared error (RMSE) of the random-effects estimates with 10% systematiclly missingness. Methods include reference (REF - before introducing missing data), complete case analysis (CCA), stratified multiple imputation (STI), multilevel multiple imputation (MLMI), and two-stage multilevel multiple imputation (2STG).

1.3 Simulation results for the continuous outcome with 30% systematically missingness

The performance of the methods is shown in Figures 6-8 for the fixed-effects parameters and in Figures 9-10 for the random-effects parameters with 30% systematically missing data.

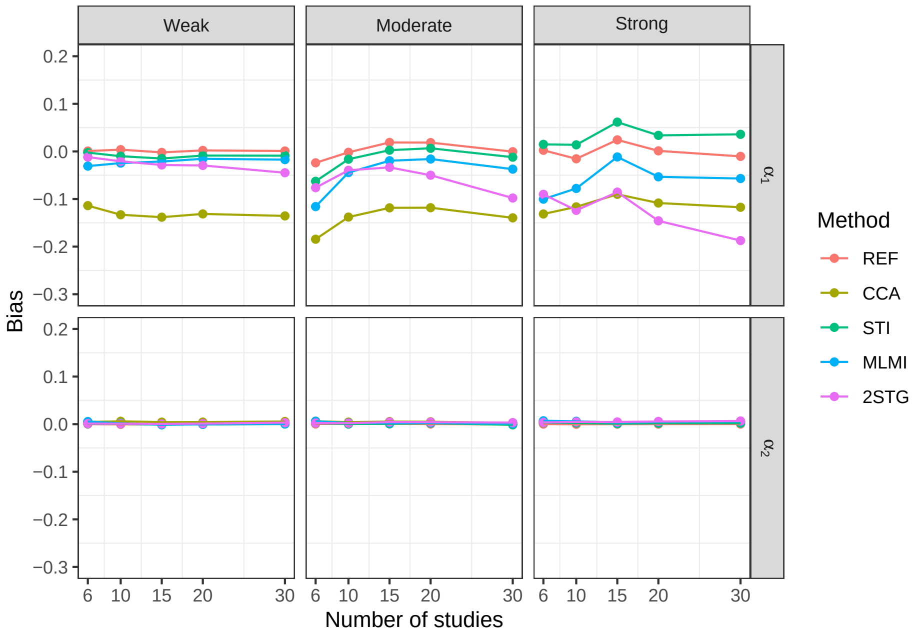

Figure 6: Bias of the fixed-effects estimates with 30% systematically missingness. Methods include reference (REF - before introducing missing data), complete case analysis (CCA), stratified multiple imputation (STI), multilevel multiple imputation (MLMI), and two-stage multilevel multiple imputation (2STG).

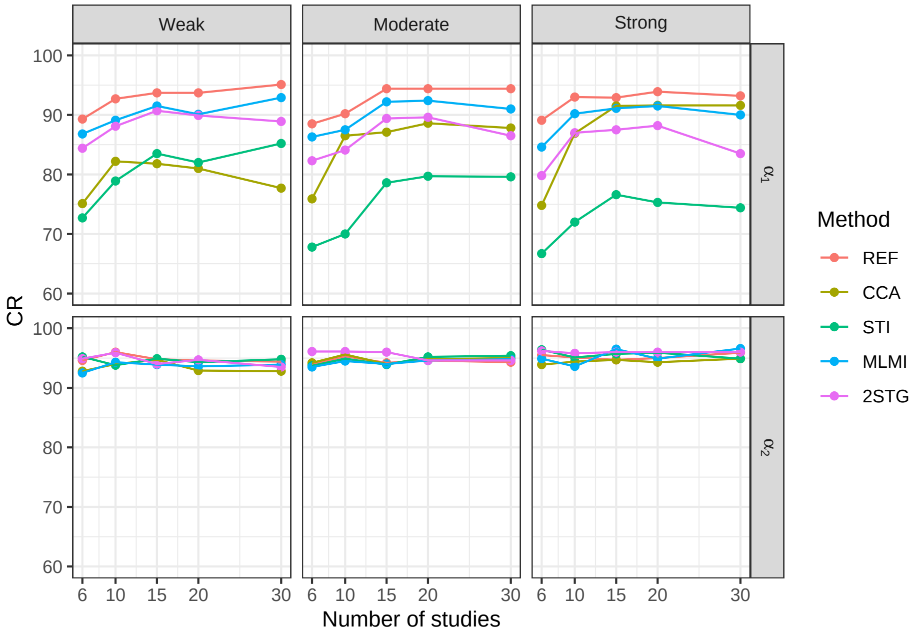

Figure 7: Coverage rate of the 95% confidence interval for the fixed-effects parameters with 30% systematically missingness. Methods include reference (REF - before introducing missing data), complete case analysis (CCA), stratified multiple imputation (STI), multilevel multiple imputation (MLMI), and two-stage multilevel multiple imputation (2STG).

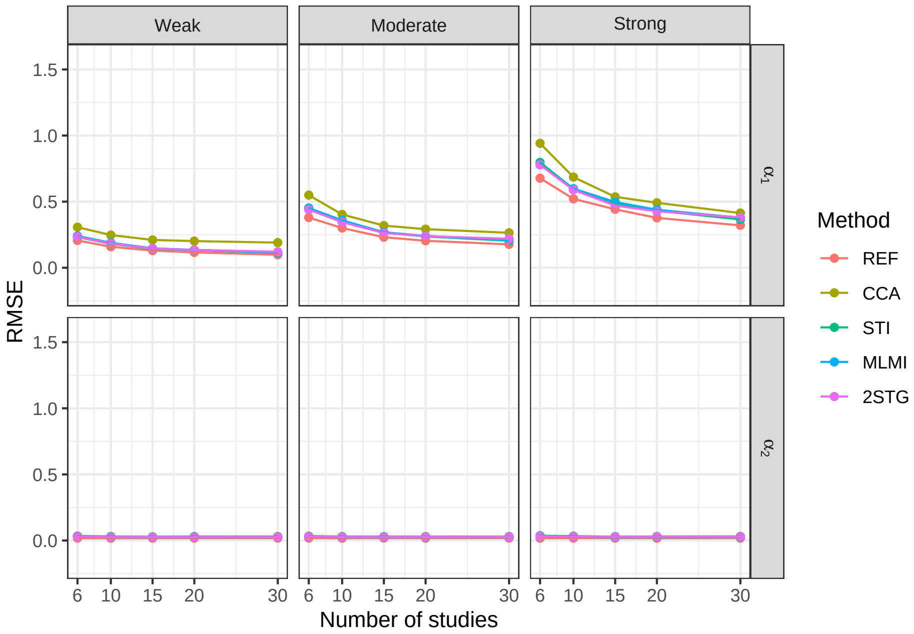

Figure 8: Root mean squared error (RMSE) of the fixed-effects estimates with 30% systematically missingness. Methods include reference (REF - before introducing missing data), complete case analysis (CCA), stratified multiple imputation (STI), multilevel multiple imputation (MLMI), and two-stage multilevel multiple imputation (2STG).

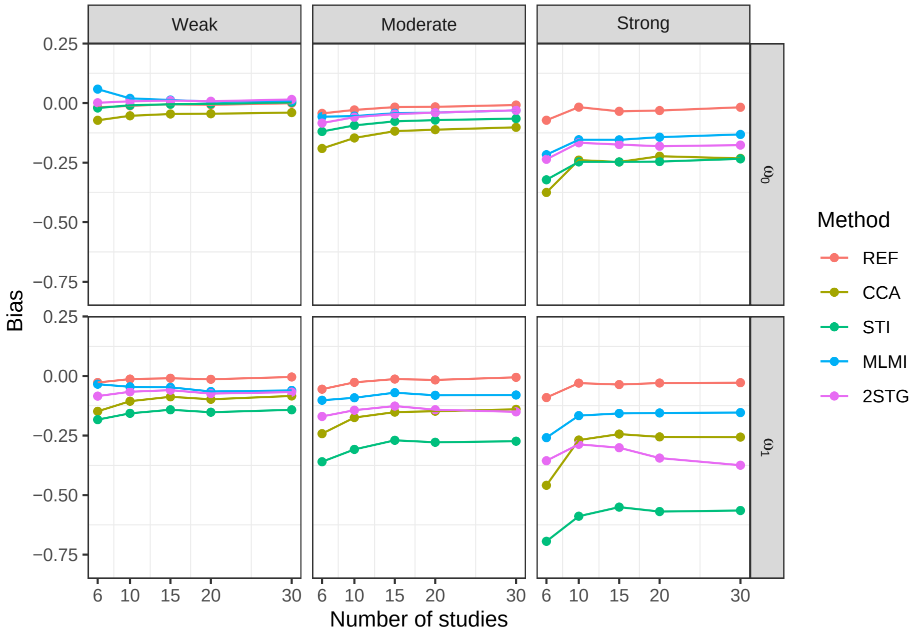

Figure 9: Bias of the random-effects estimates with 30% systematically missingness. Methods include reference (REF - before introducing missing data), complete case analysis (CCA), stratified multiple imputation (STI), multilevel multiple imputation (MLMI), and two-stage multilevel multiple imputation (2STG).

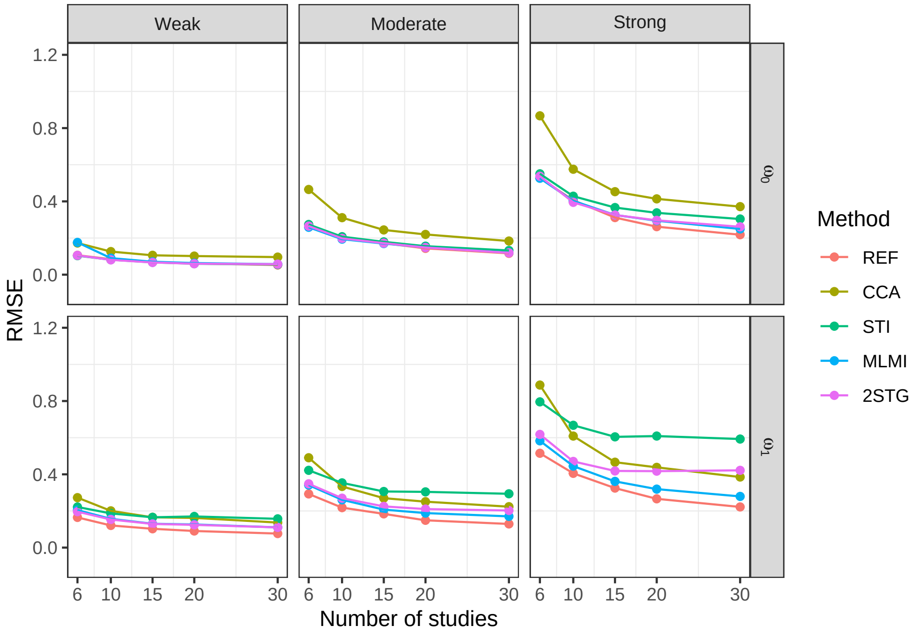

Figure 10: Root mean squared error (RMSE) of the random-effects estimates with 30% systematically missingness. Methods include reference (REF - before introducing missing data), complete case analysis (CCA), stratified multiple imputation (STI), multilevel multiple imputation (MLMI), and two-stage multilevel multiple imputation (2STG).
